# Tree biodiversity in Bornean lowland forest: What are the key species for forest city development in the new capital city of Indonesia?

**DOI:** 10.1371/journal.pone.0320489

**Published:** 2025-04-08

**Authors:** Tri Atmoko, Bina Swasta Sitepu, Wahyu Catur Adinugroho, Mukhlisi, Tri Sayektiningsih, Titiek Setyawati, Francis Q. Brearley, Ishak Yassir

**Affiliations:** 1 Research Center for Applied Zoology, Research Organization for Life Sciences and Environment, National Research and Innovation Agency (BRIN), Bogor, Indonesia; 2 Research Center for Ecology and Ethnobiology, Research Organization for Life Sciences and Environment, National Research and Innovation Agency (BRIN), Jakarta, Indonesia; 3 Makassar Environment and Forestry Standard Instrument Implementation Institute, Ministry of Environment and Forestry (BPSILHK Makassar), Makassar, Indonesia; 4 Department of Natural Sciences, Manchester Metropolitan University, Manchester, United Kingdom; 5 Samboja Environment and Forestry Standard Instrument Implementation Institute, Ministry of Environment and Forestry (BPSILHK Samboja), Samboja, Indonesia; Agriculture and Forestry University, Nepal

## Abstract

The national capital of Indonesia is in the early stages of relocation from the island of Java to East Kalimantan; Indonesia’s new capital city (Ibu Kota Negara; IKN) will be called Nusantara. The development of IKN will utilise the concept of a forest city representing the lowland forest of Borneo containing its rich biodiversity. To realize this concept, sufficient data and information regarding the status of tree diversity in this area is required. To provide this information, research was conducted in sample plots totaling 20.75 ha spread over eight locations in East Kalimantan. The selection of priority tree species for rehabilitation was carried out by using the Analytical Hierarchy Process (AHP) method with the criteria of conservation status, endemicity, climax species, distribution, food potential, ethnobotanical potential, animal food, and tree growth rates. In the sample plots, we found 5,745 trees representing 571 species with the family Dipterocarpaceae dominating, followed by Euphorbiaceae, Phyllanthaceae, Malvaceae and Annonaceae. Based on the priority categories of restoration, most of the species proposed for planting belong to the Dipterocarpaceae, in the genera *Anisoptera* (2 species), *Anthoshorea* (2 species), *Cotylelobium* (1 species), *Dipterocarpus* (3 species), *Dryobalanops* (1 species), *Hopea* (3 species), *Richetia* (3 species), *Rubroshorea* (7 species), *Shorea* (4 species), and *Vatica* (4 species). *Hopea rudiformis* is a Critically Endangered and Borneo endemic species with the highest score, and the only species included in 1^st^ priority, followed by 14 species in 2^nd^ priority, 62 species in 3^rd^ priority, and the remainder in 4^th^ priority. About 18% of tree species with potential food sources can be planted to meet the 10% target of forest cover to contribute to food security in IKN. Samboja Research Forest, Sungai Wain Protection Forest, and Bangkirai Hill are forested areas with high tree species diversity and can act as a source of seeds for the IKN nursery.

## Introduction

Borneo is one of the world’s richest areas for plant diversity [1] and home to up to 15,000 species of flowering plants [2] of which more than 1,400 are thought to be endemic [3]. Middleton et al. [4] noted that there are 3,936 endemic species in Kalimantan (Indonesian Borneo), and based on [5] it is estimated that endemic species in Borneo reach 7,000 species. Woody plant species are dominated by tree species from the Dipterocarpaceae family, which are the key family in intact forest areas [2,6–8]. The distribution of plants on the island of Borneo is strongly influenced by various environmental conditions, including the history of past climate change and current edaphic conditions [6,9,10] with the greatest plant species richness recorded in lowland forest areas [2,6].

Jakarta, Indonesia’s capital city, has become one of the most densely populated areas on the island of Java, with a population density approaching 15,978 individuals km^-2^ in 2021 [11]. Apart from being the center of government, Jakarta is also the most prominent economic center and, as with many large and developing cities, faces many environmental and social problems. These include flooding which occurs almost every year [12], piles of garbage [13], traffic jams and air pollution [14], limited supplies of clean water [15] and crowded buildings and slum settlements [16]. In addition, Jakarta is also prone to natural hazards such earthquakes, volcanoes, and even tsunamis [17].

Plans to move the Indonesian capital city from Java to Borneo have been proposed since 1957 by the first Indonesian President, Sukarno [18], and this has recently been declared by President Joko Widodo in August 2019. East Kalimantan province was chosen as the location for the new capital city because it is located centrally in the Indonesian archipelago, and this allows the change of perspective from Java-centric to Indonesia-centric. The new capital city will be called Ibu Kota Negara Nusantara or IKN. The area that has been delineated to become IKN is located in North Penajam Paser and Kutai Kartanegara regencies and is one of the hotspots of diversity for lowland plant species on the island of Borneo, especially in forest areas that are still protected [6,19–21]. Ibu Kota Negara’s land area of around 256,000 ha is currently dominated by forest cover (39%), plantations (29%), mangrove forests (2.2%), and swamp forests (1.2%) [22].

It is proposed that the new capital city will be developed by retaining the existing forest function, maintaining biodiversity, and reducing the negative impact on the environment. Ibu Kota Negara is also designated as a forest city (a city inside a forest). In the absence of a model of how to implement the forest city concept in other areas, this requires that the principles of managing biodiversity and the environment be carried out carefully [23]. The Indonesian government has set key performance indicators (KPIs) as long-term directions that guide the development and management of IKN in line with green development. One of the KPI principles is to design the city in harmony with nature, namely by developing 75% of the area with green cover (65% as protected areas and 10% to support the provision of food sources) [24].

To realize the concept of the nation’s new capital, the government will revitalize green open spaces in the core area of the capital, which is currently occupied by an industrial eucalyptus (*Eucalyptus* sp.; Myrtaceae) plantation. These non-native species will be replaced by native and local species so that they return to a landscape with a forest cover resembling native Kalimantan lowland ecosystem types. The green space target will be fulfilled through rehabilitation and reforestation, including roof gardens, subsistence farming, and botanical gardens [24]. The IKN forest rehabilitation and restoration efforts also support the international agenda to combat forest degradation and deforestation declared at the Convention on Biological Diversity COP11 in Hyderabad, India, in 2012 [25].

Rehabilitation and restoration efforts require supporting an adequate number of seed sources of species originating from the intact forest and surrounding forest areas [26]. First, we need to consider genetic diversity because it relates to forest city sustainability in the future. Low genetic diversity reduces a forest's ability to cope with diseases and extreme weather [27]. The use of local indigenous species is expected to restore the structure and composition of IKN forest tree species to mimic their original condition, especially in degraded or restored areas. Moreover, tree species to be planted must be carefully selected to create a forest with a structure and composition close to the natural conditions of Kalimantan’s existing forests. Species selection is, therefore, based on understanding the reference species found in the surrounding forest areas.

The condition of some forest areas in and around IKN remains relatively good and many studies have been carried out on their floristic structure and composition, such as the Sungai Wain Protection Forest [28], the Samboja Research Forest [19,20,29,30], Bukit Soeharto Grand Forest Park [21], PT. ITCIKU [31], and Bangkirai Hill [32,33].

Based on the above, this research aims to determine the key tree species for the development of IKN according to the concept of a forest city. The selected tree species are prioritised based on various criteria such as native species, rarity, protected status, animal food, food source, and growth rates.

## Materials and methods

### Study sites

**Fig 1:**
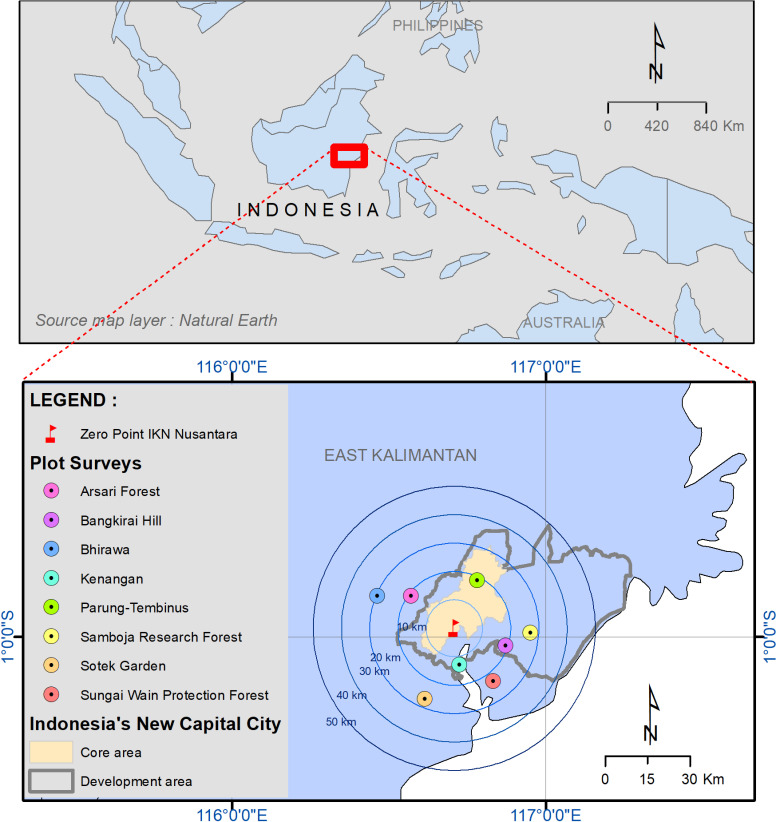
Map of research locations around Ibu Kota Negara Nusantara (Nusantara Capital City), East Kalimantan, Indonesia.

The research was conducted in forested areas within a radius of 30 km from the centre (zero point) of IKN. A total of eight sampling areas were selected from forests around IKN (Fig 1; Table 1). In each sampling area, a number of square or rectangular plots of 0.04 to 0.1 ha in size were sampled for trees ≥  10 cm diameter at breast height (dbh) (except Sotek Garden where a single plot large plot was surveyed). Field surveys in forest areas were conducted in the period 2018–2021 in the context of research activities organised by the Ministry of Environment and Forestry, Indonesia. This area remains forested (S1 Fig), although there was an area of Bangkirai Hill that experienced a fire in 1998 [33].

**Table 1:** Description of research locations around Ibu Kota Negara Nusantara, East Kalimantan, Indonesia.

Location	Coordinates	Elevation (m.a.s.l)	Number of plots	Total plot area (ha)	Date of collection	Forest type
**Arsari Forest**	S00°54’ E116°34’	385	22	1.27	2021	Secondary forest
**Bangkirai Hill**	S01°02’ E116°52’	80	48	1.92	2021	Secondary forest
**Bhirawa**	S00°52’ E116°28’	565	42	1.68	2019	Old secondary forest
**Kenangan**	S01°03’ E116°44’	5	39	2.14	2018 & 2021	Secondary forest
**Parung-Tembinus**	S00°49’ E116°47’	180	30	1.32	2021	Karst, old secondary forest
**Samboja Research Forest**	S00°59’ E116°57’	50	16	1.60	2021	Lowland tropical forest, secondary forest, swamp forest
**Sotek Garden**	S01°12’ E116°37’	20	1	10.0	2021	Old secondary forest
**Sungai Wain Protection Forest**	S01°09’ E116°50’	15	20	0.80	2019	Lowland tropical forest

The current landscape around IKN includes lowland forests, plantation forests, karst, mangrove forests, agriculture, coal mines and settlements. The topography is undulating, with elevations ranging up to 300 meters above sea level. Climatic conditions based on meteorological data collected from Sepinggan Airport, Balikpapan, show that mean annual rainfall (2001–2021) in the study location is 2,820 mm and the temperature ranges from 21.7 ºC to 35.7 ºC.

### Tree diversity

The flora recorded in the sample plots were woody trees with a diameter of more than 10 cm dbh that were identified at the Herbarium Wanariset (WAN) where needed. Relative frequency (RF), relative density (RD), and relative dominancy (Rdom) were calculated and then summed to calculate the importance value index (IVI) [34] (Eqn. 1). The vegetation in Sotek Garden was cultivated trees so, to avoid bias, the data from this location were not included in the IVI calculation; however, they were used in determining priority species.


IVI=RF+RD+Rdom (1)

Density (D) is the ratio of the number of individuals to the total area sampled, while frequency (F) is the ratio of the number of quadrats in which a species occurs to the number of quadrats observed. Thus, relative density (RD) is the density of a given species (D) divided by the density of all species (multiplied by 100) and relative frequency (RF) is the frequency of a given species (F) divided by the frequency of all species (multiplied by 100). Relative dominance (Rdom) is the basal area of a given species expressed as a percentage of the total basal area of all species present (maximum value of 100). These three measures, RF, RD, and Rdom, are summed to calculate the Importance Value Index (IVI), which lies between 0 and 300 [34].

The diversity of tree species was calculated as the Shannon-Wiener Index (*H*’). This diversity metric was calculated using R v. 3.6.3 [35] package vegan [36], provided in Eqn. 2.


$$H^{\prime }=-\overset{n}{\underset{i=1}{\sum }}\left(pi\right)log_{2}\left(pi\right)$$ (2)

Where *pi* is the proportional abundance of species *i*.

### Habitat clustering

Habitat clustering used the hierarchical clustering “ward.D” method using R v. 3.6.3 [35] package vegan [36]. Cluster distance was calculated based on the Jaccard index using the relative abundance of tree species in each location.

### Species selection

The selection of tree species for rehabilitation was carried out by scoring and weighing the criteria based on the Analytical Hierarchy Process (AHP) multi-criterion decision-making technique [37]. The publication of AHP articles is growing markedly over time with the greatest increase in the total number of articles concerning the subject area Environmental Science, probably related to the increasing interest in environmental issues in the world. However, AHP in the subject area of Ecology and Ecosystems is relatively new [38]. We applied the AHP technique using the following steps: (1) Problem Identification refers to the identification of the problem (choices) and the criteria that influence these choices, (2) Pairwise Comparison involves a pairwise comparison of the different criteria of the constructed hierarchy; the relative priorities of the criteria are calculated. (3) Eigenvector Construction and Ranking of Alternatives is the final stage in AHP. Selection was only made on trees identified to the species level (74% of all individuals). We considered eight criteria for decision making: (i) conservation status, (ii) endemicity, (iii) climax species, (iv) distribution, (v) food potential, (vi) ethnobotanical potential (mainly medicinal or cultural), (vii) animal food, and (viii) tree growth rates. Weighting of criteria used the AHP method by conducting a comparison of paired criteria [39]. Each criterion was scored against the other criteria by assigning a relative dominance value between 1 and 9 (least important to most important) based on expert opinion. Five experts in ecology, silviculture, wildlife, botany, and the environment were involved in the analysis (names provided in the Acknowledgments). In addition, these experts were also familiar with the area, the project, and the local species. Weight values were determined using the Super Decisions 2.10 program. The weight of each criterion generated from the AHP is presented in Table 2 with a consistency ratio (Cr) of 0.086 which is below the recommended maximum threshold (Cr <  0.1) [39]. The total score of each species was the sum of the eight criteria scores resulting from multiplying the weight of each criterion by the parameter scores. The list of tree species was ranked based on the total score and grouped based on the Likert scale with four categories (Priority 1^st^, 2^nd^, 3^rd^, and 4^th^) based on quartile values.

**Table 2:** Criteria weighting and scoring parameters used for the selection of tree species for rehabilitation of Ibu Kota Negara, East Kalimantan, Indonesia.

Criterion	Weight	Parameter	Score	Description	Source
Conservation status	0.292	EW, CR	3	Listed in IUCN Red List: EW (Extinct in the Wild), CR (Critically Endangered), EN (Endangered), VU (Vulnerable), NT (Near Threatened), LC (Least Concern), DD (Data Deficient), NE (Not Evaluated), - (not on the list)	www.iucnredlist.org
		EN	2		
		VU	1		
		NT, LC, DD, NE, -	0		
Endemic to Borneo	0.190	Yes	3	Endemic tree species of Borneo	[3]
		No	0		
Climax species	0.166	Yes	3	Mature tree species can occupy/form the forest canopy or emergent layer. These species belong to the Dipterocarpaceae family and are important timber species	[76]
		No	0		
Distribution	0.149	Widespread-abundant	3	The distribution of a species based on a combination of species frequency (F) and density (D) sub-scores Frequencies are Widespread, F > 0.45 (1.73); Sporadic, F 0.22-0.45 (1.15); Clustered, F < 0.22 (0.58) Densities are Abundant, D > 0.64 trees ha^-1^ (1.73); Moderate, 0.32 < D < 0.64 trees ha^-1^ (1.15); Rare, D < 0.32 trees ha^-1^ (0.58) *Note*: - frequency values based on each location - numbers in parentheses are the sub-scores	Field data
		Widespread-moderate	2		
		Widespread-rare	1		
		Sporadic-abundant	2		
		Sporadic-moderate	1.3		
		Sporadic-rare	0.7		
		Clustered-abundant	1		
		Clustered-moderate	0.7		
		Clustered-rare	0.3		
Potential as food	0.079	Yes	3	Tree species providing food sources	[77,78]
		No	0		
Potential for ethnobotanical uses	0.066	Yes	3	Tree species with medicinal uses, cultural benefits, and other benefits	[77,79]
		No	0		
Wildlife animal food	0.034	Yes	3	Flagship animal food sources. Especially for sun bears (*Helarctos malayanus*), Bucerotidae, and gibbons (*Hylobates muelleri*)	[80–84]
		No	0		
Fast-growing	0.025	Yes	3	Fast-growing species can form forest structures relatively quickly; These species are generally found in secondary forests	[43,85]
		No	0		

## Results

### Diversity of tree species

A total of 5,745 trees were recorded in the sample plots covering 571 (morpho)species, 238 genera and 81 families (Table 3). As many as 420 taxa were identified at the species level. The three locations with greatest tree densities were Parung-Tembinus, Samboja Research Forest and Bangkirai Hill, which had more than 500 trees ha^-1^. However, even though the density at Parung-Tembinus was slightly higher than in the Samboja Research Forest, the number of species found was much lower. Shannon-Wiener diversity indices for each location ranged from 1.98 to 2.87 (Table 3) with the greatest diversity found in Samboja Research Forest.

**Table 3:** Tree species richness and diversity in the eight sample locations within 30 km of Ibu Kota Negara, East Kalimantan, Indonesia.

Location	Number of trees	Density (trees ha^-1^)	Number of species	Number of genera	Number of families	Species diversity (*H*’)
Arsari Forest	619	487	230	128	56	2.31
Bangkirai Hill	962	501	179	108	50	2.50
Bhirawa	435	258	126	84	48	2.12
Kenangan	719	332	128	82	40	1.98
Parung-Tembinus	701	531	137	91	41	2.22
Samboja Research Forest	843	526	212	137	53	2.87
Sotek Garden	1,080	108	83	56	32	2.47
Sungai Wain Protection Forest	386	482	155	95	41	2.63
All locations	5,745	277	571	238	81	2.39

### Clustering by location

**Fig 2:**
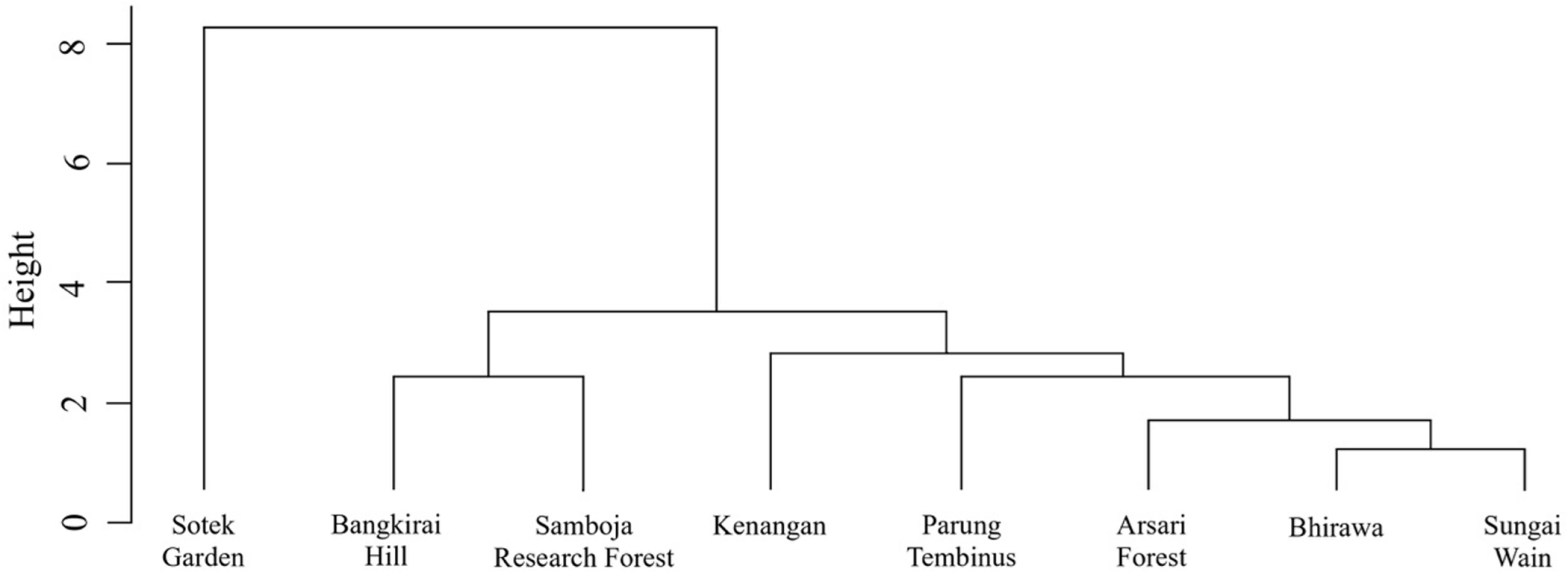
Habitat cluster dendrogram based on binary data on the presence of tree species in eight forest locations within 30 km of Ibu Kota Negara, East Kalimantan, Indonesia.

The eight study locations are divided into two main clusters (Fig 2). Sotek Gardens formed a separate cluster because it is a former community settlement that has not been occupied for a long time, and has developed into an old secondary forest dominated by large-diameter fruit trees. The other seven locations are natural and old secondary forests, and some are burnt areas. The forests in the Bhirawa and Sungai Wain Protection Forests had similar tree species. The Bhirawa sample plot was a production forest area protected by the company as a source of *Shorea* (and other Dipterocarpaceae) seeds. Sungai Wain was also a protected forest area, so both are relatively safe from disturbances. The forests in Bangkirai Hill and Samboja Research Forest had similar tree species composition because they were relatively close together and the sample plots included areas of forest that were still in good condition, in addition to forests that had been burnt.

### Species composition

**Fig 3:**
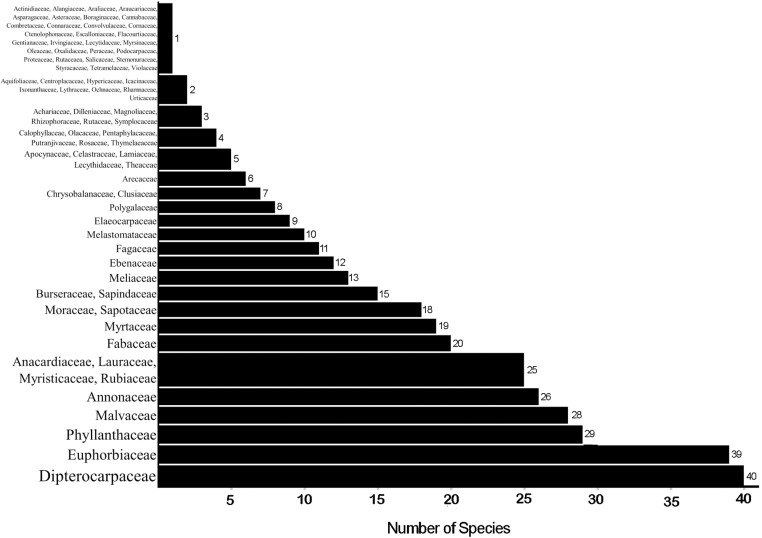
The families and number of species in each family at eight forest locations within 30 km of Ibu Kota Negara, East Kalimantan, Indonesia.

The results showed that the family with the greatest number of species found in the forests around IKN was the Dipterocarpaceae, followed by Euphorbiaceae, Malvaceae, and Phyllanthaceae (Fig 3). Four hundred and fifty-eight individual trees (40 species) were within the Dipterocarpaceae from 11 genera, namely: *Anisoptera* (2 species), *Anthoshorea* (2 species), *Cotylelobium* (1 species), *Dipterocarpus* (5 species), *Dryobalanops* (1 species), *Hopea* (4 species), *Parashorea* (1 species), *Richetia* (3 species), *Rubroshorea* (8 species), *Shorea* (4 species), and *Vatica* (8 species).

Jambu-jambu (*Syzygium* sp.; Myrtaceae) and large-leaved mahang (*Macaranga gigantea*; Euphorbiaceae) had the greatest IVI values among 571 species found at the study location although it is quite likely that *Syzygium* sp. included more than one species (Table 4). These species are common in lowland secondary forest habitats as pioneer species along with laban (*Vitex pinnata*; Lamiaceae) and *Strobocalyx arborea* (Asteraceae). Three species of *Rubroshorea* were recorded among the ten species with the greatest IVI values. *Rubroshorea* also occupied the top position among the IVIs for genera. These results showed that the study location is comprised of a mix of secondary forests (from various histories) as well as areas that are less disturbed and are typical lowland evergreen rain forest, a.k.a. mixed dipterocarp forest (MDF).

**Table 4:** The top ten species, genera and families based on IVI in the forest locations in the vicinity of Ibu Kota Negara, East Kalimantan, Indonesia.

Species	IVI	Genus	IVI	Family	IVI
*Syzygium* sp.	6.52	*Rubroshorea*	28.32	Dipterocarpaceae	34.57
*Macaranga gigantea*	5.29	*Macaranga*	15.98	Euphorbiaceae	21.81
*Rubroshorea parvifolia*	5.12	*Syzygium*	11.99	Lauraceae	15.55
*Eusideroxylon zwageri*	4.60	*Artocarpus*	7.76	Myrtaceae	14.05
*Strobocalyx arborea*	4.49	*Litsea*	5.21	Moraceae	11.94
*Rubroshorea ovalis*	4.08	*Lithocarpus*	4.91	Phyllanthaceae	9.67
*Rubroshorea smithiana*	3.57	*Eusideroxylon*	4.85	Annonaceae	9.12
*Vitex pinnata*	3.20	*Madhuca*	4.80	Myristicaceae	9.04
*Madhuca kingiana*	3.07	*Pternandra*	4.71	Malvaceae	9.01
*Schima wallichii*	3.04	*Strobocalyx*	4.71	Sapotaceae	8.90

### Species categories

Most tree species were fast-growing and are a source of animal food (S1 Table). One hundred and twenty-three tree species were known to have potential social and economic functions for the people of Borneo, both as a source of food and medicine. Based on the IUCN Red List categorisation, 11.7% of the species were threatened, one of which (*Mangifera casturi*; Anacardiaceae) was declared Extinct in the Wild. This species was only found as a cultivated fruit tree at Sotek Garden. All Critically Endangered species (8) belonged to the Dipterocarpaceae, namely *Anthoshorea lamellata*, *Dipterocarpus cornutus*, *D. kunstleri*, *Hopea mengarawan*, *H. rudiformis*, *Rubroshorea johorensis*, *Shorea inappendiculata*, and *Vatica venulosa*. The Dipterocarpaceae also dominated as the climax species by contributing 40 species out of a total of 97 climax species found at the study site. A total of seven Endangered species were found: *Agathis borneensis* (Araucariaceae), *Anisoptera costata* (Dipterocarpaceae), *Dipterocarpus grandiflorus* (Dipterocarpaceae), *Santiria rubiginosa* (Burseraceae), *Rubroshorea pauciflora* (Dipterocarpaceae), *Shorea dasyphylla* (Dipterocarpaceae), and *Syzygium scortechinii*, while there were 33 species in the Vulnerable category. Only one species was found which is legally protected under Indonesian regulations, namely bandang (*Borassodendron borneense*; Arecaceae), as one of the endemic palms of Borneo.

### Species selection for rehabilitation

Species selection for rehabilitation was based on criteria including conservation status, endemism, climax species, distribution, potential as a food source, ethnobotanical uses, key animal food sources, and fast-growing species. Among 420 species (S1 Table), we found one species as the first priority for rehabilitation followed by 15 species, 89 species, and 315 species for the second, third, and fourth priorities, respectively (Table 5).

**Table 5:** The top fifty priority tree species for rehabilitation and reforestation of Ibu Kota Negara, East Kalimantan, Indonesia based upon eight criteria included in an AHP analysis.

Rank	Species	Total Score	Priority
1	*Hopea rudiformis*	2,043	1^st^
2	*Mangifera casturi*	1,835	2^nd^
3	*Durio kutejensis* ^*^	1,549	2^nd^
4	*Anthoshorea lamellata*, *Hopea mengarawan*, *Rubroshorea johorensis*	1,473	2^nd^
5	*Rubroshorea smithiana*	1,459	2^nd^
6	*Santiria rubiginosa*	1,432	2^nd^
7	*Dipterocarpus cornutus*, *Dipterocarpus. kunstleri*, *Shorea inappendiculata*, *Vatica venulosa*	1,424	2^nd^
8	*Anthoshorea ochracea*, *Richetia macrobalanos*, *Richetia mujongensis*, *Vatica sarawakensis*	1,410	2^nd^
9	*Artocarpus tamaran*	1,300	3^rd^
10	*Palaquium sericeum*	1,295	3^rd^
11	*Pentace laxiflora*	1,293	3^rd^
12	*Ficus variegata* ^*^	1,260	3^rd^
13	*Durio dulcis*, *Mangifera pajang*	1,251	3^rd^
14	*Eusideroxylon zwageri* ^*^	1,240	3^rd^
15	*Durio lanceolatus*	1,220	3^rd^
16	*Dracontomelon dao*, *Pometia pinnata*	1,209	3^rd^
17	*Pentace erectinervia*	1,193	3^rd^
18	*Durio zibethinus*	1,185	3^rd^
19	*Dipterocarpus grandiflorus*	1,181	3^rd^
20	*Dipterocarpus confertus*, Dryobalanops lanceolata^*^, *Rubroshorea parvistipulata*	1,167	3^rd^
21	*Pterospermum javanicum*	1,160	3^rd^
22	*Scorodocarpus borneensis*	1,134	3^rd^
23	*Agathis borneensis*, *Anisoptera costata*, *Rubroshorea pauciflora*, *Shorea dasyphylla*	1,132	3^rd^
24	*Richetia patoiensis*, *Vatica oblongifolia*	1,118	3^rd^
25	*Alseodaphne elmeri*	1,063	3^rd^
26	*Artocarpus anisophyllus*	1,054	3^rd^
27	*Borassodendron borneense*	1,008	3^rd^
28	*Dialium platysepalum*	0,987	3^rd^
29	*Aquilaria microcarpa* ^*^	0,964	3^rd^
30	*Parkia timoriana*	0,962	3^rd^
31	*Horsfieldia reticulata*, *Sandoricum borneense*	0,959	3^rd^
32	*Myristica simiarum*	0,942	3^rd^
33	*Shorea seminis*	0,936	3^rd^
34	*Gonystylus consanguineous*, *Horsfieldia gracilis*	0,912	3^rd^
35	*Aporosa nitida*, *Litsea umbellata*	0,897	3^rd^
36	*Madhuca kingiana*	0,896	3^rd^
37	*Shorea laevis* ^*^	0,889	3^rd^
38	*Madhuca motleyana*, *Payena acuminata*, *Pterospermum diversifolium*	0,887	3^rd^
39	*Syzygium tawahense*	0,846	3^rd^
40	*Anisoptera marginata*, *Hopea beccariana*, *Rubroshorea balangeran*, *Vatica pauciflora*	0,840	3^rd^
41	*Dyera costulata*, *Lagerstroemia speciosa*, *Peronema canescens*	0,821	3^rd^
42	*Artocarpus integer*, *Nephelium lappaceum*	0,809	3^rd^
43	*Cotylelobium melanoxylon*, *Rubroshorea ovalis*	0,795	3^rd^
44	*Gymnacranthera farquhariana*, *Litsea elliptica*	0,774	3^rd^
45	*Knema pallens*, *Knema percoriacea*, *Myristica villosa*, *Palaquium stenophyllum*	0,771	3^rd^
46	*Artocarpus rigidus*	0,762	3^rd^
47	*Lithocarpus gracilis*	0,750	3^rd^
48	*Macaranga beccariana*	0,744	3^rd^
49	*Durio oxleyanus*	0,730	3^rd^
50	*Rubroshorea leprosula* ^*^	0,723	3^rd^

Remarks:

* Seed sources are available in IKN.

## Discussion

### Status of tree species diversity

Biodiversity is crucial to support human well-being and to provide life-supporting services such as clean water, food, pollination, climate control, and flood prevention. It also plays roles in improving quality of life and human health and prosperity [40,41,42]. Therefore, biodiversity should become the main indicator for successful forest city development in IKN. This research found that the landscape around the proposed IKN contained high tree species richness, particularly in less-disturbed forested areas such as Samboja Research Forest and Sungai Wain Protection Forest. Previous research also found that Samboja Research Forest had the second highest plant diversity across forests in Indonesia with 553 identified tree species [20]. Of the 13 Dipterocarpaceae genera found in Kalimantan, only the monospecific genera *Neohopea* and *Upuna* were not found in this study area. However, there are records from Kartawinata et al. [20] of the presence of *Upuna* at Samboja Research Forest based on an inventory in 2004.

Whilst the Dipterocarpaceae was the dominant family as expected [6,7,8], the Euphorbiaceae, Malvaceae and Phyllanthaceae were also abundant in the landscape. These families are known to be characteristic of lowland evergreen rain forest in Kalimantan. In a rehabilitation context, fast-growing pioneer tree species (such as members of the Euphorbiaceae) can make the micro-climate shadier, thus allowing better growth of shade-tolerant species [43,44]. The pioneer tree species with short lifespans live up to around 30 years [45], then allowing shade-tolerant species to form the subsequent canopy.

### Priority species

The development of IKN is within the framework of a forest city, namely a city designed on a landscape basis placing forest ecosystems at the centre of urban spatial planning and facilitating the life of urban communities [46]. To revitalize green open areas and to implement the forest city concept, replacement of *Eucalyptus* spp. with native species from Borneo is needed. The high score given to conservation status and endemicity shows the spirit of IKN development by prioritizing rare and endangered tree species to support recovery of Borneo’s tropical forests. To rehabilitate forest ecosystems, appropriate species selection is fundamental to its success. Species selected should be native [47,48,49] and have high survival rates [50]. In addition, fast-growing species can be added to the species selected, but we should pay attention to the trade-offs related to life-history strategies since fast-growing species have short lifespans [51]. The advantage of planting fast-growing species in the initial step of rehabilitation is the ability of these species to rapidly form canopy cover [47], thereby creating microclimates suitable for the growth of shade-tolerant species as noted above. Several studies recommend combining trees with a diversity of traits in rehabilitation, such as pioneer species and climax species [52], or fast-growing native tree species and slow-growing native tree species [49]. There is also increasing recognition that tree selection should be not only be considered in terms of ecosystem recovery but also for local communities. Therefore, selected trees should have economic benefits and improve local livelihoods [27,53,54].

The overlap of tree species that produce food for humans and food for animals has the potential to cause conflict if not managed properly. This is because all potential species providing food sources for humans also provide food sources for animals, especially primates. Some primates present in the study area that have the potential to cause conflict with humans are long-tailed monkeys (*Macaca fascicularis*), macaques (*Macaca nemestrina*), proboscis monkeys (*Nasalis larvatus*) and orangutans (*Pongo pygmaeus*). The first three species have natural distribution in the study area, while orangutans have been introduced to Sungai Wain Protection Forest and Gunung Beratus Protection Forest.

The prioritization of species to be planted based on the regional spatial plan of IKN [55] must be adjusted to the characteristics and suitability of the growing area. Our study did not include mangrove or wetland habitats, and tree selection was based on the terrestrial characteristics of the study area. Emphasis on the right trees for the right place and the right purposes is the main underlying challenge that needs to be tackled to make restoration through tree-based interventions successful [56]. Species in priority 1 and 2 can be used for species enrichment or collection gardens (S1 Table) as a demonstration for the use of key species in the IKN region. Due to the complexity of environments and site diversity, testing tree species under local biotic and management conditions to identify their adaptations to site variables is essential. We recommend that future studies or restoration plans in the region include soil assessments (soil physico-chemical properties) to guide species selection and improve the success of rehabilitation efforts.

### Seed source availability

Seed source availability is essential to developing the forest city [57,58]. There are seven seed sources currently available, which includes the *Rubroshorea leprosula* seed source at Samboja Research Forest with a total production of 5 ha, plus *Dipterocarpus humeratus* (5 ha) and *Eusideroxylon zwageri* (Lauraceae) (30 ha). Other seed sources are available for *Aquilaria microcarpa* (Thymelaeaceae) (5 ha), *Agathis borneensis* (5 ha) and *Durio kutejensis* (Malvaceae) (2.5 ha) [59], whilst *Ficus variegata* (Moraceae) (5 ha), Tengkawang (*Rubroshorea stenoptera*) (3 ha), *Rubroshorea ovalis* (1 ha), Tengkawang (*Rubroshorea beccariana*) (6 ha), Kapur (*Dryobalanops beccarii*) (5 ha) and *Shorea laevis* (5 ha) have been growing since 2015 (Nanang Riana, Manager of Samboja Research Forest, pers. comm.). Another approach to obtain seeds is buying from seed industries or local communities’ nurseries. Seeds used for rehabilitation should have good performance, so it is important to understand the seeds’ origin. Indeed, seeds derived from seed industries have to be accompanied by a seed certificate [60]. Furthermore, buying seeds from local communities is another option to supply the seed requirements for rehabilitation. Some people living in the Samboja area have developed seed nurseries for personal and commercial purposes, and supporting these growers will provide economic benefits to them. This activity can stimulate the local economy by creating job opportunities such as seed collectors and nursery caretakers, increasing communities’ income. For rehabilitation purposes, the government has established a large nursery that produces 15 million seeds per annum in Mentawir, Penajam Paser Utara district.

An obstacle emerges to provide seed sources for high-scoring rare species where there is limited information on their ecology and thus silvicultural techniques. There are some efforts to overcome this challenge by natural collection and vegetative propagation. Vegetative propagation, tissue culture and shoot cuttings may be alternatives to supply seeds through generative techniques [61–65]. We therefore suggest that future restoration efforts incorporate propagation research and the development of local nurseries to ensure that the selected species can be effectively propagated and thus out-planted.

### Rehabilitation strategies

The development of IKN needs to integrate environmental and forestry issues, including wildlife home ranges and habitat protection within the landscape. Currently, forest plantations of *Eucalyptus* spp. cover a considerable proportion of the landscape within IKN. Nevertheless, they will be replaced by native and endemic species of lowland tropical forest of Kalimantan as we discuss here.

There are four strands encompassing successful strategies for rehabilitation in order to achieve the target of green cover of 75%, which are forest rehabilitation, land rehabilitation, reclamation, and social forestry (Table 6). Rehabilitation and reclamation are defined according to Peraturan Pemerintah No. 26/2020 [66] on Forest Rehabilitation and Reclamation. Initially, forest rehabilitation aims to recover degraded forest. This can then be expanded to other issues, since factors contributing to forest degradation are complex. Forest rehabilitation is now not only considered from the point-of-view of forest recovery but also that of local prosperity. Nawir et al. [67] offer recommendations in implementing successful rehabilitation: (1) good knowledge on rehabilitation sites (soils, topography, local culture, local wisdom), (2) seed source availability, (3) local community participation, (4) project sustainability, and (5) clear land tenure.

**Table 6:** Rehabilitation strategies in the proposed Ibu Kota Negara area, East Kalimantan, Indonesia.

Strategy	Objective	Coverage	Implementation
Forest Rehabilitation	Restore, preserve, and increase forest function to improve carrying capacity, productivity, and functions to maintain life support systems	Conservation forest (except Nature Reserve and National Park core zone), Production Forest, and Protection Forest	- Intensive reforestation (Critical land with zero community activity) - Agroforestry (Critical land with community activity)
Land Rehabilitation	Restore, preserve, and increase land function to improve carrying capacity, productivity, and functions to maintain life support systems	Outside forest area with forest cover or land area	Revegetation with the scheme: - Private forest - Environmental revegetation - Urban Forest (Settlement area, industrial area, recreation area, biodiversity sanctuary area, protection area)
Forest Reclamation	To improve or restore the degraded forest area to functioning optimally and according to the designation	Degraded forest with land and vegetation change due to utilization	Revegetation with tree plantations and maintenance in the post-use forest to restore and recover degraded vegetation
Social Forestry	Local communities as a principal element in the sustainable forest management to improve prosperity, environmental balance, and social/cultural dynamics	State Forest, Private Forest, and Indigenous Forest	Village Forest, Indigenous Forest, Community Forest, Community Plantation Forest, and Forestry/Conservation Partnership

Source: [66].

The development of IKN needs to integrate environmental and forestry issues, including wildlife home range and habitat protection within the landscape. Based on Law No. 3 of 2022 concerning the National Capital, 75% of the area will be maintained as green space (65% forest +  10% food source plants). The regional spatial plan of IKN [53] allocates protected areas in IKN as a component of the forest city implementation. The five types of protected areas in the spatial plans are: environmental buffer areas and food security; local protected areas (protection to coastlines, riverbanks, and around lakes or reservoirs); green open areas (urban forest, city park, sub-district park, village park, green belt, burial ground); conservation area; and mangrove ecosystems.

The Urban Forest covers an area of 64,618 ha, most of which will be developed from the former concession area of plantations of *Eucalyptus* spp. Not all parts of the site will be used to build infrastructure facilities; however, most will be rehabilitated to restore the *Eucalyptus* plantation to the original tropical rain forest using the native and endemic species of the lowland tropical forest of Borneo. The urban forest also includes two wildlife corridors that will be built in the IKN area, namely the northern corridor connecting Bukit Suharto Grand Forest Park (GFP) with the Production Forest and the southern corridor connecting Bukit Suharto GFP with Sungai Wain Protection Forest.

Bukit Suharto GFP is the only conservation area in IKN that covers lowland forest areas to the coast, including the unique heath forest ecosystem. The area of 64,255 ha is not entirely forest; forest fires have damaged part of it, and some of it has been converted into agricultural fields and is occupied by the local community. This area will be maintained and restored to become a key part of the identity of the forest city. Hajjar et al. [68] stated that community participation will improve their responsibility and commitment to preserve the forest. This involvement should start from the project plan and design to the program’s implementation, including the methods used in forest management and species selection for rehabilitation activities. In Indonesia, Safe’i et al. [69] demonstrated that a social forestry program with a community forest scheme in Tanggamus Regency, Lampung (Sumatra), contributed to the economic improvement of the forest farmer group by about 15%. Another community’s forest scheme also contributed to the recovery of the forest area of about 29.3% from 40 ha of the project area in Gunung Kidul Regency, Yogyakarta [70]. Several factors that contribute to social forestry programs’ success include technical and financial assistance for forest farmers in forest management and product processing [71], and their capacity building [72].

Land rehabilitation, forest reclamation, and social forestry could all use species as food source trees to meet 10% of the targeted green cover in IKN. Nevertheless, the choice of food source trees needs to be considered with the involvement of local communities who have knowledge of the species’ attributes in terms of economic benefits, medicinal use, and social/cultural values [73]. The introduction of poorly-known species in community participation projects is possible as long as this is followed by supporting activities in the appropriate planting techniques, maintenance, harvesting, post-harvest production, and marketing strategies.

As 10% of the green cover is allocated for food security, the zoning of species planted in land rehabilitation activities is important to avoid animal-human conflict. Planting animal food source species should be done in forest edge areas or restored forest areas. The species selected for planting in these areas are those with potential for animals but low preference for humans. Meanwhile, human food source species should be planted away from forest boundaries or around settlements. To mitigate the risk of conflict, we propose a spatial zoning approach in the city forest design where trees that serve as animal food sources are planted in areas further from human settlements and city centers, creating natural buffers and wildlife corridors. This would encourage wildlife to remain in designated forest areas while minimizing direct interactions with the urban population. Additionally, as part of the forest city planning, monitoring and management strategies should be developed to address potential human-wildlife interactions proactively.

Forest rehabilitation in the IKN area also could be conducted via natural regeneration [74] or secondary forest succession. In particular, for the *Eucalyptus* plantations, the canopy cover could be opened to facilitate the climax species seed germination and initiate appropriate microclimate conditions as shown by Brancallion et al. [75] in Brazil. Removal of *Eucalyptus* close to the natural forest border is also essential to facilitate the natural regeneration of natural forest plant species. There are some examples of this processes in other tropical regions, but limited guidance for restoration of *Eucalyptus* plantations into natural dipterocarp forest leads to a challenging opportunity.

## Conclusions

Rehabilitation activities in the IKN area towards natural ecosystem conditions can be fulfilled by selecting and using priority tree species based on the natural habitats of these species in Borneo. Species selection should be further conducted according to the availability of seeds and the availability of rehabilitation areas in order to support the formation of the lowland evergreen (mixed dipterocarp) rain forest ecosystem as the original and natural ecosystem in the IKN area. This ecosystem recovery strategy requires several critical stages considering the area’s biophysical conditions and the surrounding communities’ socio-economic conditions. Choosing the right rehabilitation strategies will help achieve the goals of developing the IKN within the forest city concept and have a positive impact on the surrounding community during urban development.

## Supporting information

S1 Data Data IKN. (XLSX)

S1 Fig Land use/land cover map (2023) around Ibu Kota Negara Nusantara (Nusantara Capital City), East Kalimantan, Indonesia. (JPG)

S1 Table List of trees in 20.75 ha of plots in eight locations around Ibu Kota Negara, East Kalimantan, Indonesia. (DOCX)
