# Supplementary figures and images for: Tree biodiversity in Bornean lowland forest: What are the key species for forest city development in the new capital city of Indonesia?
Source: PLoS One. 2025 Apr 8;20(4):e0320489. doi: 10.1371/journal.pone.0320489 (PMC11978104; doi:10.1371/journal.pone.0320489)

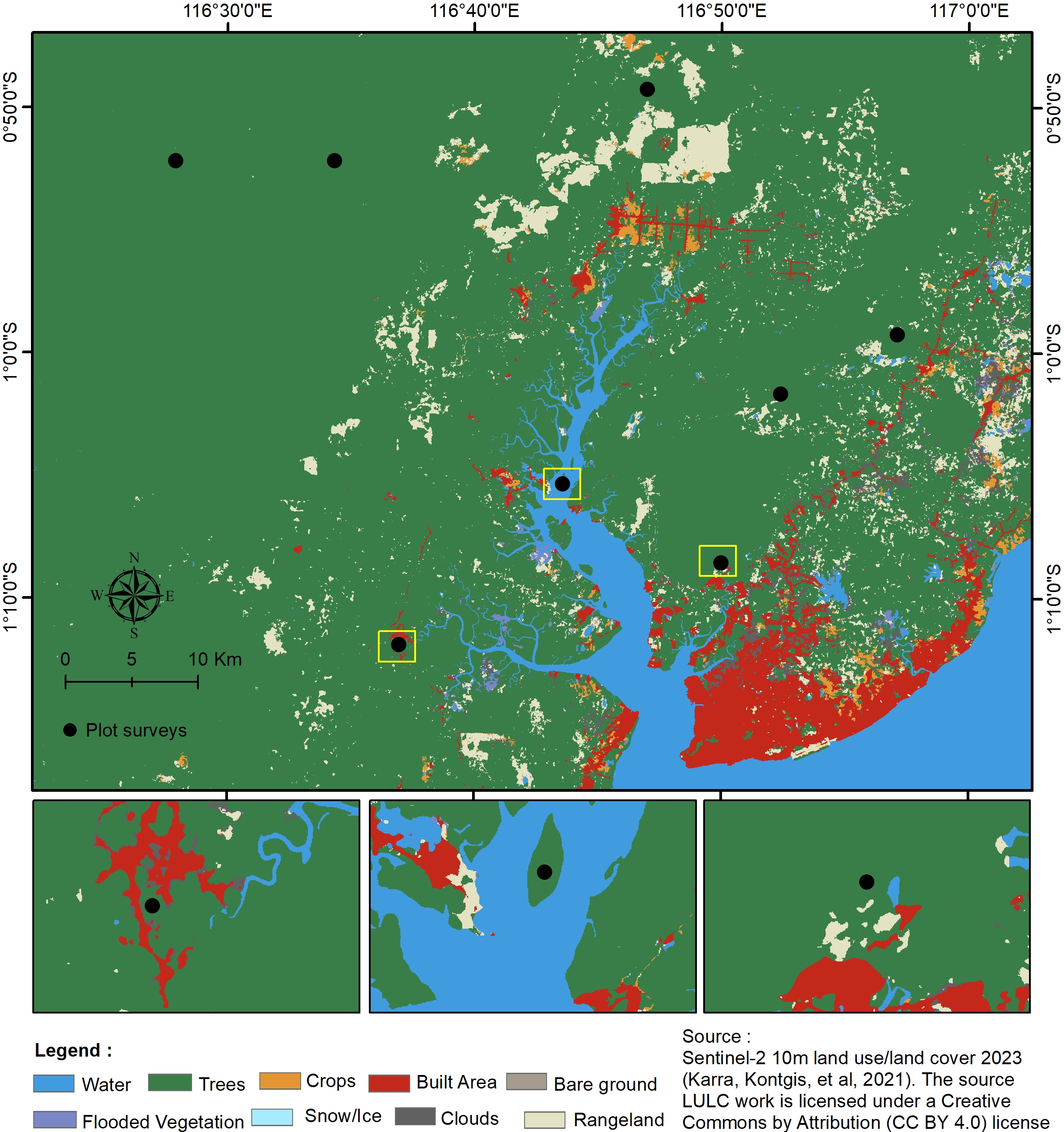

Supplement: S1 Fig — (JPG) [file pone.0320489.s002.jpg]
