# Supplementary material for: Tree biodiversity in Bornean lowland forest: What are the key species for forest city development in the new capital city of Indonesia?
Source: PLoS One. 2025 Apr 8;20(4):e0320489. doi: 10.1371/journal.pone.0320489 (PMC11978104; doi:10.1371/journal.pone.0320489)
Supplement: S1 Table — (DOCX) [file pone.0320489.s003.docx]

**Table S1. List of trees in 20.75 ha of plots in eight locations around Ibu Kota Negara, East Kalimantan, Indonesia**.

| No | Family | Species | Conservation status | Borneo Endem | Climax species | Food source | Medicine & culture | Animal Food | Fast Growing | Distribution | Suggestions for planting purposes | | | |
| --- | --- | --- | --- | --- | --- | --- | --- | --- | --- | --- | --- | --- | --- | --- |
|  |  |  |  |  |  |  |  |  |  |  | Rehabilitation | Reclamation | Social Foretry | Park/ Recreation/ Roadside |
| 1 | Achariaceae | *Hydnocarpus polypetalus* (Slooten) Sleumer | - | - | - | - | - | ✔️ | - | Rare-Sporadic | ✔️ | - | - | - |
| 2 | Achariaceae | *Ryparosa kostermansii* Sleumer | - | ✔️ | - | - | - | - | - | Rare-Clustery | ✔️ | - | - | - |
| 3 | Actinidiaceae | *Saurauia javanica* (Blume ex Nees) Hoogland | - | - | - | ✔️ | - | ✔️ | - | Rare-Clustery | ✔️ | - | ✔️ | ✔️ |
| 4 | Alangiaceae | *Alangium javanicum* (Blume) Wang | LC | - | - | - | - | - | ✔️ | Rare-Sporadic | ✔️ | ✔️ | - | - |
| 5 | Anacardiaceae | *Bouea oppositifolia* (Roxb.) Meisn. | LC | - | - | - | - | - | - | Rare-Clustery | ✔️ | - | - | - |
| 6 | Anacardiaceae | *Buchanania arborescens* (Blume) Blume | LC | - | - | - | ✔️ | - | - | Rare-Clustery | ✔️ | - | ✔️ | - |
| 7 | Anacardiaceae | *Buchanania sessifolia* Blume | LC | - | - | ✔️ | ✔️ | ✔️ | ✔️ | Rare-Sporadic | ✔️ | ✔️ | ✔️ | - |
| 8 | Anacardiaceae | *Campnosperma auriculatum* (Blume) Hook.f. | LC | - | - | - | - | - | - | Rare-Clustery | ✔️ | - | - | - |
| 9 | Anacardiaceae | *Dracontomelon dao* (Blanco) Merr. & Rolfe | LC | - | ✔️ | ✔️ | ✔️ | ✔️ | ✔️ | Rare-Sporadic | ✔️ | ✔️ | ✔️ | - |
| 10 | Anacardiaceae | *Drimycarpus luridus* (Hook.f.) Ding Hou | - | - | - | - | - | - | - | Rare-Sporadic | ✔️ | - | - | - |
| 11 | Anacardiaceae | *Gluta macrocarpa* (Engl.) Ding Hou | - | - | - | - | - | - | - | Rare-Widely spread | ✔️ | - | - | - |
| 12 | Anacardiaceae | *Gluta renghas* L. | NT | - | ✔️ | - | - | - | ✔️ | Rare-Clustery | ✔️ | ✔️ | ✔️ | - |
| 13 | Anacardiaceae | *Gluta wallichii* (Hook.f.) Ding Hou | LC | - | - | - | - | - | ✔️ | Rare-Sporadic | ✔️ | ✔️ | - | - |
| 14 | Anacardiaceae | *Mangifera caesia* Jack | NT | - | - | ✔️ | ✔️ | ✔️ | - | Rare-Clustery | ✔️ | - | ✔️ | - |
| 15 | Anacardiaceae | *Mangifera casturi* Kosterm. | EW | ✔️ | - | ✔️ | - | ✔️ | - | Rare-Clustery | ✔️ | - | ✔️ | - |
| 16 | Anacardiaceae | *Mangifera foetida* Lour. | LC | - | - | ✔️ | ✔️ | ✔️ | - | Rare-Clustery | ✔️ | - | ✔️ | - |
| 17 | Anacardiaceae | *Mangifera indica* L. | DD | - | - | ✔️ | ✔️ | ✔️ | - | Rare-Clustery | ✔️ | - | ✔️ | - |
| 18 | Anacardiaceae | *Mangifera pajang* Kosterm. | VU | ✔️ | - | ✔️ | - | ✔️ | - | Rare-Clustery | ✔️ | - | ✔️ | - |
| 19 | Anacardiaceae | *Mangifera similis* Blume | VU | - | - | - | - | ✔️ | - | Rare-Clustery | ✔️ | - | - | - |
| 20 | Anacardiaceae | *Melanochyla bullata* Ding Hou | LC | ✔️ | - | - | - | - | ✔️ | Rare-Clustery | ✔️ | ✔️ | - | - |
| 21 | Anacardiaceae | *Melanochyla fulvinervis* (Blume) Ding Hou | - | - | - | - | - | - | ✔️ | Rare-Clustery | ✔️ | ✔️ | - | - |
| 22 | Anacardiaceae | *Pentaspadon motleyi* Hook. F. | DD | - | - | ✔️ | ✔️ | ✔️ | - | Rare-Clustery | ✔️ | - | ✔️ | - |
| 23 | Annonaceae | *Cananga odorata* (Lam.) Hook.f.& Thomson | LC | - | - | - | ✔️ | ✔️ | ✔️ | Moderate-Widely spread | ✔️ | ✔️ | ✔️ | ✔️ |
| 24 | Annonaceae | *Goniothalamus ridleyi* King | - | - | - | - | - | - | - | Rare-Clustery | ✔️ | - | - | - |
| 25 | Annonaceae | *Huberantha rumphii* (Blume ex Hensch.) Chaowasku | - | - | - | - | - | - | ✔️ | Rare-Sporadic | ✔️ | ✔️ | - | ✔️ |
| 26 | Annonaceae | *Mezzettia parviflora* Becc. | - | - | - | - | - | - | - | Rare-Clustery | ✔️ | - | - | - |
| 27 | Annonaceae | *Monocarpia euneura* Miq. | VU | - | - | - | - | - | ✔️ | Rare-Clustery | ✔️ | ✔️ | - | ✔️ |
| 28 | Annonaceae | *Monocarpia kalimantanensis* P.J.A.Kessler | LC | ✔️ | - | - | - | ✔️ | - | Rare-Clustery | ✔️ | - | - | ✔️ |
| 29 | Annonaceae | *Maasia glauca* (Hassk.) Mols, Kessler & Rogstad | LC | - | - | - | - | ✔️ | - | Rare-Clustery | ✔️ | - | - | ✔️ |
| 30 | Annonaceae | *Maasia sumatrana* (Miq.) Mols, Kessler & Rogstad | LC | - | - | - | - | - | ✔️ | Rare-Sporadic | ✔️ | ✔️ | - | ✔️ |
| 31 | Annonaceae | *Monoon lateriflorum* (Blume) Miq. | - | - | - | - | - | - | - | Rare-Widely spread | ✔️ | - | - | - |
| 32 | Annonaceae | *Popowia pisocarpa* (Blume) Endl. | - | - | - | - | - | - | ✔️ | Rare-Clustery | ✔️ | ✔️ | - | ✔️ |
| 33 | Annonaceae | *Pseuduvaria pamattonis* (Miq.) Y.C.F.Su & R.M.K.Saunders | LC | - | - | - | - | - | - | Rare-Clustery | ✔️ | - | - | - |
| 34 | Annonaceae | *Stelechocarpus cauliflorus* (Scheff.) R.E.Fr. | - | - | - | - | - | - | - | Rare-Clustery | ✔️ | - | - | - |
| 35 | Annonaceae | *Xylopia elliptica* Maingay ex Hook.f. & Thomson | LC | - | - | - | - | ✔️ | - | Rare-Sporadic | ✔️ | - | - | - |
| 36 | Annonaceae | *Xylopia ferruginea* Baill. | - | - | - | - | - | ✔️ | ✔️ | Rare-Sporadic | ✔️ | ✔️ | - | - |
| 37 | Annonaceae | *Xylopia malayana* Hook.f. & Thomson | LC | - | - | - | - | ✔️ | - | Rare-Sporadic | ✔️ | - | - | - |
| 38 | Annonaceae | *Xylopia sumatrana*(Miq.) D.M.Johnson & N.A.Murray | - | - | - | - | - | ✔️ | - | Rare-Clustery | ✔️ | - | - | - |
| 39 | Apocynaceae | *Alstonia pneumatophora* Backer ex Den Berger | LC | - | - | - | - | - | ✔️ | Rare-Clustery | ✔️ | ✔️ | - | ✔️ |
| 40 | Apocynaceae | *Alstonia iwahigensis* Elmer | - | - | - | - | ✔️ | - | ✔️ | Rare-Widely spread | ✔️ | ✔️ | ✔️ | ✔️ |
| 41 | Apocynaceae | *Dyera costulata* Hook.f. | LC | - | ✔️ | - | ✔️ | - | ✔️ | Rare-Clustery | ✔️ | ✔️ | ✔️ | ✔️ |
| 42 | Apocynaceae | *Willughbeia coriacea*Wall. | LC | - | - | - | ✔️ | ✔️ | ✔️ | Rare-Clustery | ✔️ | ✔️ | ✔️ | ✔️ |
| 43 | Apocynaceae | *Willughbeia sarawacensis* (Pierre) K.Schum. | - | - | - | ✔️ | - | ✔️ | - | Rare-Clustery | ✔️ | - | ✔️ | ✔️ |
| 44 | Aquifoliaceae | *Ilex cymosa* Blume | LC | - | - | - | ✔️ | ✔️ | ✔️ | Rare-Sporadic | ✔️ | ✔️ | ✔️ | - |
| 45 | Araliaceae | *Polyscias diversifolia* (Blume) Lowry & G.M.Plunkett | LC | - | - | - | - | - | - | Rare-Clustery | ✔️ | - | - | - |
| 46 | Araucariaceae | *Agathis borneensis* Warb. | EN | - | ✔️ | - | - | - | - | Rare-Clustery | ✔️ | - | ✔️ | ✔️ |
| 47 | Arecaceae | *Areca catechu* L. | - | - | - | ✔️ | ✔️ | ✔️ | ✔️ | Rare-Clustery | ✔️ | ✔️ | ✔️ | ✔️ |
| 48 | Arecaceae | *Arenga pinnata* (Wurmb) Merr. | - | - | - | ✔️ | ✔️ | ✔️ | - | Rare-Clustery | ✔️ | - | ✔️ | ✔️ |
| 49 | Arecaceae | *Borassodendron borneense* J.Dransf. | - | ✔️ | - | ✔️ | - | ✔️ | - | Rare-Sporadic | ✔️ | - | ✔️ | ✔️ |
| 50 | Arecaceae | *Caryota mitis* Lour. | LC | - | - | ✔️ | - | ✔️ | - | Rare-Clustery | ✔️ | - | ✔️ | ✔️ |
| 51 | Arecaceae | *Oncosperma horridum* (Griff.) Scheff. | - | - | - | ✔️ | - | ✔️ | - | Rare-Widely spread | ✔️ | - | ✔️ | ✔️ |
| 52 | Arecaceae | *Pholidocarpus majadum* Becc. | LC | - | - | - | - | - | - | Rare-Sporadic | ✔️ | - | - | - |
| 53 | Asparagaceae | *Dracaena angustifolia* (Medik.) Roxb. | - | - | - | - | ✔️ | - | - | Rare-Clustery | ✔️ | - | ✔️ | ✔️ |
| 54 | Asteraceae | *Strobocalyx arborea* (Buch.-Ham.) Sch.Bip. | - | - | - | - | ✔️ | - | ✔️ | Moderate-Widely spread | ✔️ | ✔️ | ✔️ | - |
| 55 | Boraginaceae | *Pteleocarpa lamponga* (Miq.) Bakh. | LC | - | - | - | - | - | - | Rare-Clustery | ✔️ | - | - | - |
| 56 | Burseraceae | *Dacryodes rugosa* (Blume) H.J.Lam | LC | - | - | ✔️ | - | ✔️ | ✔️ | Rare-Sporadic | ✔️ | ✔️ | ✔️ | - |
| 57 | Burseraceae | *Dacryodes rostrata* (Blume) H.J.Lam | LC | - | - | ✔️ | - | ✔️ | - | Rare-Widely spread | ✔️ | - | ✔️ | - |
| 58 | Burseraceae | *Dacryodes costata* (A.W.Benn.) H.J.Lam | LC | - | - | ✔️ | - | ✔️ | - | Rare-Clustery | ✔️ | - | ✔️ | - |
| 59 | Burseraceae | *Canarium denticulatum* Blume | - | - | - | - | - | ✔️ | - | Rare-Clustery | ✔️ | - | - | - |
| 60 | Burseraceae | *Canarium grandifolium* (Ridl.) H.J.Lam | - | - | - | - | - | - | - | Rare-Clustery | ✔️ | - | - | - |
| 61 | Burseraceae | *Canarium littorale* Blume | LC | - | - | - | ✔️ | ✔️ | - | Rare-Clustery | ✔️ | - | ✔️ | - |
| 62 | Burseraceae | *Canarium pilosum* A.W.Benn. | - | - | - | - | - | - | - | Rare-Clustery | ✔️ | - | - | - |
| 63 | Burseraceae | *Santiria griffithii* (Hook.f.) Engl. | LC | - | - | - | - | ✔️ | - | Rare-Clustery | ✔️ | - | - | - |
| 64 | Burseraceae | *Santiria oblongifolia* Blume | - | - | - | - | - | ✔️ | - | Rare-Clustery | ✔️ | - | - | - |
| 65 | Burseraceae | *Santiria rubiginosa* Blume | EN | - | ✔️ | - | ✔️ | ✔️ | - | Rare-Clustery | ✔️ | - | ✔️ | - |
| 66 | Burseraceae | *Santiria tomentosa* Blume | LC | - | - | ✔️ | ✔️ | ✔️ | - | Rare-Clustery | ✔️ | - | ✔️ | - |
| 67 | Burseraceae | *Triomma malaccensis* Hook.f. | - | - | ✔️ | - | - | - | - | Rare-Clustery | ✔️ | - | ✔️ | - |
| 68 | Calophyllaceae | *Calophyllum depressinervosum* M.R.Hend. & Wyatt-Sm. | - | - | ✔️ | - | - | - | - | Rare-Clustery | ✔️ | - | ✔️ | - |
| 69 | Calophyllaceae | *Calophyllum nodosum* Vesque | - | - | - | - | - | - | - | Rare-Clustery | ✔️ | - | - | - |
| 70 | Calophyllaceae | *Kayea borneensis* P.F.Stevens | - | ✔️ | - | - | - | - | - | Rare-Clustery | ✔️ | - | - | - |
| 71 | Calophyllaceae | *Mammea acuminata* Kosterm. | LC | ✔️ | - | - | - | - | ✔️ | Rare-Clustery | ✔️ | ✔️ | - | - |
| 72 | Cannabaceae | *Gironniera nervosa* Planch. | - | - | ✔️ | - | - | - | ✔️ | Rare-Sporadic | ✔️ | ✔️ | ✔️ | - |
| 73 | Celastraceae | *Bhesa paniculata* Arn. | LC | - | - | - | ✔️ | - | ✔️ | Rare-Sporadic | ✔️ | ✔️ | ✔️ | - |
| 74 | Celastraceae | *Lophopetalum javanicum* (Zoll.) Turcz. | LC | - | ✔️ | - | - | - | - | Rare-Clustery | ✔️ | - | ✔️ | - |
| 75 | Celastraceae | *Lophopetalum pallidum* M.A.Lawson | - | - | ✔️ | - | - | - | - | Rare-Clustery | ✔️ | - | ✔️ | - |
| 76 | Centroplacaceae | *Bhesa robusta* (Roxb.) Ding Hou | LC | - | - | - | - | - | - | Rare-Clustery | ✔️ | - | - | - |
| 77 | Chrysobalanaceae | *Angelesia splendens* Korth. | LC | - | - | - | - | - | - | Rare-Sporadic | ✔️ | - | - | - |
| 78 | Chrysobalanaceae | *Atuna excelsa* (Jack) Kosterm. | - | - | - | - | - | - | - | Rare-Clustery | ✔️ | - | - | - |
| 79 | Chrysobalanaceae | *Atuna racemosa* Raf. | LC | - | ✔️ | - | - | - | ✔️ | Rare-Clustery | ✔️ | ✔️ | ✔️ | - |
| 80 | Chrysobalanaceae | *Maranthes corymbosa* Blume | LC | - | ✔️ | - | - | - | ✔️ | Rare-Clustery | ✔️ | ✔️ | ✔️ | - |
| 81 | Chrysobalanaceae | *Parinari oblongifolia* Hook.f. | - | - | - | - | - | - | ✔️ | Rare-Clustery | ✔️ | ✔️ | - | - |
| 82 | Clusiaceae | *Garcinia mangostana* L. | - | - | - | ✔️ | ✔️ | ✔️ | - | Rare-Clustery | ✔️ | - | ✔️ | - |
| 83 | Clusiaceae | *Garcinia nervosa* Miq. | - | - | - | - | - | ✔️ | - | Rare-Clustery | ✔️ | - | - | - |
| 84 | Clusiaceae | *Garcinia nigrolineata* Planch. ex T.Anderson | - | - | - | - | - | - | - | Rare-Clustery | ✔️ | - | - | - |
| 85 | Clusiaceae | *Garcinia parvifolia* (Miq.) Miq. | - | - | - | ✔️ | ✔️ | ✔️ | - | Rare-Sporadic | ✔️ | - | ✔️ | - |
| 86 | Combretaceae | *Terminalia foetidissima* Griff. | LC | - | - | - | - | - | ✔️ | Rare-Sporadic | ✔️ | ✔️ | - | - |
| 87 | Connaraceae | *Ellipanthus beccarii* Pierre | - | - | - | - | - | - | - | Rare-Clustery | ✔️ | - | - | - |
| 88 | Ctenolophonaceae | *Ctenolophon parvifolius* Oliv. | VU | - | - | - | - | - | - | Rare-Clustery | ✔️ | - | - | - |
| 89 | Dilleniaceae | *Dillenia excelsa* (Jack) Martelli ex Gilg. | - | - | - | - | ✔️ | - | ✔️ | Rare-Clustery | ✔️ | ✔️ | ✔️ | ✔️ |
| 90 | Dilleniaceae | *Dillenia grandifolia* Wall. ex Hook.f. & Thomson | - | - | ✔️ | - | - | - | - | Rare-Clustery | ✔️ | - | ✔️ | - |
| 91 | Dilleniaceae | *Dillenia reticulata* King | LC | - | - | - | - | - | ✔️ | Rare-Sporadic | ✔️ | ✔️ | - | - |
| 92 | Dipterocarpaceae | *Anisoptera costata* Korth. | EN | - | ✔️ | - | - | - | - | Rare-Clustery | ✔️ | - | ✔️ | - |
| 93 | Dipterocarpaceae | *Anisoptera marginata* Korth. | VU | - | ✔️ | - | - | - | - | Rare-Clustery | ✔️ | - | ✔️ | - |
| 94 | Dipterocarpaceae | *Anthoshorea lamellata*(Foxw.) P.S.Ashton & J.Heck. | CR | - | ✔️ | - | - | - | - | Rare-Sporadic | ✔️ | - | ✔️ | - |
| 95 | Dipterocarpaceae | *Anthoshorea ochracea*(Symington) P.S.Ashton & J.Heck. | VU | ✔️ | ✔️ | - | - | - | - | Rare-Clustery | ✔️ | - | ✔️ | - |
| 96 | Dipterocarpaceae | *Cotylelobium melanoxylon* (Hook.f.) Pierre | LC | - | ✔️ | - | ✔️ | - | - | Rare-Sporadic | ✔️ | - | ✔️ | - |
| 97 | Dipterocarpaceae | *Dipterocarpus confertus* Sloot. | NT | ✔️ | ✔️ | - | - | - | - | Rare-Sporadic | ✔️ | - | ✔️ | - |
| 98 | Dipterocarpaceae | *Dipterocarpus cornutus* Dyer | CR | - | ✔️ | - | - | - | - | Rare-Clustery | ✔️ | - | ✔️ | - |
| 99 | Dipterocarpaceae | *Dipterocarpus grandiflorus* (Blanco) Blanco | EN | - | ✔️ | - | - | - | - | Rare-Sporadic | ✔️ | - | ✔️ | - |
| 100 | Dipterocarpaceae | *Dipterocarpus humeratus* Slooten | NT | - | ✔️ | - | - | - | - | Rare-Clustery | ✔️ | - | ✔️ | - |
| 101 | Dipterocarpaceae | *Dipterocarpus kunstleri* King | CR | - | ✔️ | - | - | - | - | Rare-Clustery | ✔️ | - | ✔️ | - |
| 102 | Dipterocarpaceae | *Dryobalanops lanceolata* Burck | LC | ✔️ | ✔️ | - | - | - | - | Rare-Sporadic | ✔️ | - | ✔️ | - |
| 103 | Dipterocarpaceae | *Hopea beccariana* Burck | VU | - | ✔️ | - | - | - | - | Rare-Clustery | ✔️ | - | ✔️ | - |
| 104 | Dipterocarpaceae | *Hopea dryobalanoides* Miq. | LC | - | ✔️ | - | - | - | - | Rare-Clustery | ✔️ | - | ✔️ | - |
| 105 | Dipterocarpaceae | *Hopea mengarawan* Miq. | CR | - | ✔️ | - | - | - | - | Rare-Sporadic | ✔️ | - | ✔️ | - |
| 106 | Dipterocarpaceae | *Hopea rudiformis* P.S. Ashton | CR | ✔️ | ✔️ | - | - | - | - | Rare-Sporadic | ✔️ | - | ✔️ | - |
| 107 | Dipterocarpaceae | *Parashorea malaanonan* (Blanco) Merr. | LC | - | ✔️ | - | - | - | - | Rare-Clustery | ✔️ | - | ✔️ | - |
| 108 | Dipterocarpaceae | *Richetia macrobalanos*(P.S.Ashton) P.S.Ashton & J.Heck. | VU | ✔️ | ✔️ | - | - | - | - | Rare-Clustery | ✔️ | - | ✔️ | - |
| 109 | Dipterocarpaceae | *Richetia mujongensis*(P.S.Ashton) P.S.Ashton & J.Heck. | Vu | ✔️ | ✔️ | - | - | - | - | Rare-Clustery | ✔️ | - | ✔️ | - |
| 110 | Dipterocarpaceae | *Richetia patoiensis*(P.S.Ashton) P.S.Ashton & J.Heck. | NT | ✔️ | ✔️ | - | - | - | - | Rare-Clustery | ✔️ | - | ✔️ | - |
| 111 | Dipterocarpaceae | *Rubroshorea balangeran* (Korth.) P.S.Ashton & J.Heck. | VU | - | ✔️ | - | - | - | - | Rare-Clustery | ✔️ | - | ✔️ | - |
| 112 | Dipterocarpaceae | *Rubroshorea dasyphylla* (Foxw.) P.S.Ashton & J.Heck. | EN | - | ✔️ | - | - | - | - | Rare-Clustery | ✔️ | - | ✔️ | - |
| 113 | Dipterocarpaceae | *Rubroshorea johorensis*(Foxw.) P.S.Ashton & J.Heck. | CR | - | ✔️ | - | - | - | - | Rare-Sporadic | ✔️ | - | ✔️ | - |
| 114 | Dipterocarpaceae | *Rubroshorea leprosula*(Miq.) P.S.Ashton & J.Heck. | NT | - | ✔️ | - | - | - | ✔️ | Rare-Widely spread | ✔️ | ✔️ | ✔️ | - |
| 115 | Dipterocarpaceae | *Rubroshorea ovalis*(Korth.) P.S.Ashton & J.Heck | LC | - | ✔️ | - | ✔️ | - | - | Rare-Sporadic | ✔️ | - | ✔️ | - |
| 116 | Dipterocarpaceae | *Rubroshorea parvifolia*(Dyer) P.S.Ashton & J.Heck | LC | - | ✔️ | - | - | - | - | Rare-Widely spread | ✔️ | - | ✔️ | - |
| 117 | Dipterocarpaceae | *Rubroshorea parvistipulata*(F.Heim) P.S.Ashton & J.Heck. | LC | ✔️ | ✔️ | - | - | - | - | Rare-Sporadic | ✔️ | - | ✔️ | - |
| 118 | Dipterocarpaceae | *Rubroshorea pauciflora*(King) P.S.Ashton & J.Heck. | EN | - | ✔️ | - | - | - | - | Rare-Clustery | ✔️ | - | ✔️ | - |
| 119 | Dipterocarpaceae | *Rubroshorea smithiana*(Symington) P.S.Ashton & J.Heck. | VU | ✔️ | ✔️ | - | - | - | - | Rare-Sporadic | ✔️ | - | ✔️ | - |
| 120 | Dipterocarpaceae | *Shorea inappendiculata* Burck. | CR | - | ✔️ | - | - | - | - | Rare-Clustery | ✔️ | - | ✔️ | - |
| 121 | Dipterocarpaceae | *Shorea laevis* Ridl. | VU | - | ✔️ | - | - | - | - | Rare-Sporadic | ✔️ | - | ✔️ | - |
| 122 | Dipterocarpaceae | *Shorea seminis* (de Vriese) Slooten | LC | - | ✔️ | ✔️ | - | ✔️ | - | Rare-Sporadic | ✔️ | - | ✔️ | - |
| 123 | Dipterocarpaceae | *Vatica oblongifolia* Hook.f. | LC | ✔️ | ✔️ | - | - | - | - | Rare-Clustery | ✔️ | - | ✔️ | - |
| 124 | Dipterocarpaceae | *Vatica odorata* (Griff.) Symington | - | - | ✔️ | - | - | - | - | Rare-Clustery | ✔️ | - | ✔️ | - |
| 125 | Dipterocarpaceae | *Vatica pauciflora* (Korth.) Blume | Vu | - | ✔️ | - | - | - | - | Rare-Clustery | ✔️ | - | ✔️ | - |
| 126 | Dipterocarpaceae | *Vatica rassak* (Korth.) Blume | LC | - | ✔️ | - | - | - | - | Rare-Clustery | ✔️ | - | ✔️ | - |
| 127 | Dipterocarpaceae | *Vatica sarawakensis* F.Heim | VU | ✔️ | ✔️ | - | - | - | - | Rare-Clustery | ✔️ | - | ✔️ | - |
| 128 | Dipterocarpaceae | *Vatica umbonata* (Hook.f.) Burck | LC | - | ✔️ | - | - | - | - | Rare-Sporadic | ✔️ | - | ✔️ | - |
| 129 | Dipterocarpaceae | *Vatica venulosa* Blume | CR | - | ✔️ | - | - | - | - | Rare-Clustery | ✔️ | - | ✔️ | ✔️ |
| 130 | Ebenaceae | *Diospyros armata*Hemsl. | - | - | - | - | - | ✔️ | - | Rare-Clustery | ✔️ | - | - | - |
| 131 | Ebenaceae | *Diospyros borneensis* Hiern | LC | - | - | - | ✔️ | ✔️ | ✔️ | Rare-Widely spread | ✔️ | ✔️ | ✔️ | - |
| 132 | Ebenaceae | *Diospyros buxifolia* (Blume) Hiern | - | - | - | - | - | ✔️ | - | Rare-Clustery | ✔️ | - | - | ✔️ |
| 133 | Ebenaceae | *Diospyros confertiflora* (Hiern) Bakh. | - | - | - | - | - | ✔️ | ✔️ | Rare-Sporadic | ✔️ | ✔️ | - | - |
| 134 | Ebenaceae | *Diospyros elliptifolia* Merr. | - | - | - | - | - | ✔️ | ✔️ | Rare-Clustery | ✔️ | ✔️ | - | - |
| 135 | Ebenaceae | *Diospyros oblonga*Wall. ex G.Don | - | - | ✔️ | - | - | ✔️ | - | Rare-Sporadic | ✔️ | - | ✔️ | - |
| 136 | Ebenaceae | *Diospyros sumatrana* Miq. | DD | - | - | - | - | ✔️ | ✔️ | Rare-Sporadic | ✔️ | ✔️ | - | - |
| 137 | Elaeocarpaceae | *Elaeocarpus griffithii* (Wright) A.Gray | - | - | - | - | - | - | ✔️ | Rare-Clustery | ✔️ | ✔️ | - | - |
| 138 | Elaeocarpaceae | *Elaeocarpus kostermansii* Weibel | - | ✔️ | - | - | - | - | - | Rare-Clustery | ✔️ | - | - | - |
| 139 | Elaeocarpaceae | *Elaeocarpus macrocerus* (Turcz.) Merr. | LC | - | - | - | - | - | ✔️ | Rare-Clustery | ✔️ | ✔️ | - | - |
| 140 | Elaeocarpaceae | *Elaeocarpus stipularis* Blume | - | - | - | - | ✔️ | - | ✔️ | Rare-Sporadic | ✔️ | ✔️ | ✔️ | - |
| 141 | Elaeocarpaceae | *Elaeocarpus valetonii* Hochr. | LC | - | - | - | - | - | ✔️ | Rare-Clustery | ✔️ | ✔️ | - | - |
| 142 | Elaeocarpaceae | *Sloanea javanica* (Miq.) Szyszyl. ex K.Schum. | - | - | ✔️ | - | - | - | - | Rare-Clustery | ✔️ | - | ✔️ | - |
| 143 | Euphorbiaceae | *Acalypha caturus* Blume | LC | - | - | - | ✔️ | - | ✔️ | Rare-Clustery | ✔️ | ✔️ | ✔️ | - |
| 144 | Euphorbiaceae | *Blumeodendron subrotundifolium* (Elmer) Merr. | LC | - | - | - | - | - | - | Rare-Clustery | ✔️ | - | - | - |
| 145 | Euphorbiaceae | *Croton argyratus* Blume | LC | - | - | - | ✔️ | - | ✔️ | Rare-Widely spread | ✔️ | ✔️ | ✔️ | - |
| 146 | Euphorbiaceae | *Croton griffithii* Hook.f. | - | - | - | - | - | - | ✔️ | Rare-Sporadic | ✔️ | ✔️ | - | - |
| 147 | Euphorbiaceae | *Endospermum diadenum* (Miq.) Airy Shaw | LC | - | - | - | ✔️ | - | ✔️ | Rare-Sporadic | ✔️ | ✔️ | ✔️ | - |
| 148 | Euphorbiaceae | *Endospermum peltatum* Merr. | LC | - | - | - | - | - | ✔️ | Rare-Clustery | ✔️ | ✔️ | - | - |
| 149 | Euphorbiaceae | *Erismanthus obliquus* Wall. ex Müll.Arg. | - | - | - | - | - | - | - | Rare-Clustery | ✔️ | - | - | - |
| 150 | Euphorbiaceae | *Hancea penangensis* (Müll.Arg.) S.E.C.Sierra, Kulju & Welzen | LC | - | - | - | - | - | ✔️ | Rare-Sporadic | ✔️ | ✔️ | - | - |
| 151 | Euphorbiaceae | *Macaranga depressa* (Müll.Arg.) Müll.Arg. | - | - | - | - | - | - | ✔️ | Rare-Clustery | ✔️ | ✔️ | - | - |
| 152 | Euphorbiaceae | *Macaranga bancana* (Miq.) Müll.Arg. | - | - | - | - | ✔️ | - | ✔️ | Rare-Sporadic | ✔️ | ✔️ | ✔️ | - |
| 153 | Euphorbiaceae | *Macaranga beccariana* Merr. | LC | ✔️ | - | - | - | - | ✔️ | Rare-Sporadic | ✔️ | ✔️ | - | - |
| 154 | Euphorbiaceae | *Macaranga conifera* (Zoll.) Müll.Arg. | - | - | - | - | ✔️ | - | ✔️ | Rare-Sporadic | ✔️ | ✔️ | ✔️ | - |
| 155 | Euphorbiaceae | *Macaranga gigantea* (Reichb.f. & Zoll.) Müll.Arg. | - | - | - | - | ✔️ | ✔️ | ✔️ | Abundance-Sporadic | ✔️ | ✔️ | ✔️ | - |
| 156 | Euphorbiaceae | *Macaranga hypoleuca* (Reichb.f. & Zoll.) Müll.Arg. | - | - | - | - | ✔️ | - | ✔️ | Rare-Widely spread | ✔️ | ✔️ | ✔️ | - |
| 157 | Euphorbiaceae | *Macaranga lowii* King ex Hook.f. | LC | - | - | - | - | - | ✔️ | Rare-Clustery | ✔️ | ✔️ | - | - |
| 158 | Euphorbiaceae | *Macaranga motleyana* (Müll.Arg.) Müll.Arg. | - | - | - | - | ✔️ | - | ✔️ | Rare-Sporadic | ✔️ | ✔️ | ✔️ | - |
| 159 | Euphorbiaceae | *Macaranga pearsonii* Merr. | LC | ✔️ | - | - | - | - | ✔️ | Rare-Clustery | ✔️ | ✔️ | - | - |
| 160 | Euphorbiaceae | *Macaranga pruinosa* (Miq.) Müll.Arg. | - | - | - | - | - | ✔️ | ✔️ | Rare-Sporadic | ✔️ | ✔️ | - | - |
| 161 | Euphorbiaceae | *Macaranga tanarius* (L.) Müll.Arg. | LC | - | - | - | ✔️ | - | ✔️ | Rare-Clustery | ✔️ | ✔️ | ✔️ | - |
| 162 | Euphorbiaceae | *Mallotus dispar* (Blume) Müll.Arg. | LC | - | ✔️ | - | - | - | ✔️ | Rare-Clustery | ✔️ | ✔️ | ✔️ | - |
| 163 | Euphorbiaceae | *Mallotus lackeyi* Elmer | LC | - | - | - | - | - | ✔️ | Rare-Clustery | ✔️ | ✔️ | - | - |
| 164 | Euphorbiaceae | *Mallotus paniculatus* (Lam.) Müll.Arg. | LC | - | - | - | ✔️ | - | ✔️ | Rare-Clustery | ✔️ | ✔️ | ✔️ | - |
| 165 | Euphorbiaceae | *Micrococca oligandra*(Müll.Arg.) Prain Endl. ex Hassk. | LC | - | - | - | - | - | ✔️ | Rare-Clustery | ✔️ | ✔️ | - | - |
| 166 | Euphorbiaceae | *Moultonianthus leembruggianus* (Boerl. & Koord.) Steenis | - | - | - | - | - | - | - | Rare-Clustery | ✔️ | - | - | - |
| 167 | Euphorbiaceae | *Neoscortechinia kingii* (Hook.f.) Pax & K.Hoffm. | - | - | - | - | - | - | - | Rare-Clustery | ✔️ | - | - | - |
| 168 | Euphorbiaceae | *Paracroton pendulus* (Hassk.) Miq. | LC | - | - | - | - | - | ✔️ | Rare-Sporadic | ✔️ | ✔️ | - | - |
| 169 | Euphorbiaceae | *Pimelodendron griffithianum* (Müll.Arg.) Benth. ex Hook.f. | - | - | - | - | - | - | - | Rare-Clustery | ✔️ | - | - | - |
| 170 | Euphorbiaceae | *Ptychopyxis bacciformis* Croizat | - | - | - | - | - | - | - | Rare-Clustery | ✔️ | - | - | - |
| 171 | Euphorbiaceae | *Trigonostemon laevigatus* Müll.Arg. | LC | - | ✔️ | - | - | - | - | Rare-Clustery | ✔️ | - | ✔️ | - |
| 172 | Fabaceae | *Archidendron jiringa* (Jack) I.C.Nielsen | - | - | - | ✔️ | ✔️ | ✔️ | ✔️ | Rare-Clustery | ✔️ | ✔️ | ✔️ | - |
| 173 | Fabaceae | *Archidendron clypearia* (Jack.) I.C.Nielsen | LC | - | - | - | - | - | ✔️ | Rare-Sporadic | ✔️ | ✔️ | - | ✔️ |
| 174 | Fabaceae | *Archidendron microcarpum* (Benth.) I.C.Nielsen | - | - | - | - | ✔️ | - | ✔️ | Rare-Clustery | ✔️ | ✔️ | ✔️ | ✔️ |
| 175 | Fabaceae | *Crudia bantamensis* (Hassk.) Benth. | - | - | - | - | - | - | - | Rare-Clustery | ✔️ | - | - | - |
| 176 | Fabaceae | *Dialium indum* L. | - | - | - | ✔️ | - | ✔️ | - | Rare-Sporadic | ✔️ | - | ✔️ | - |
| 177 | Fabaceae | *Dialium platysepalum* Baker | - | - | ✔️ | ✔️ | - | ✔️ | - | Rare-Widely spread | ✔️ | - | ✔️ | - |
| 178 | Fabaceae | *Fordia splendidissima* (Miq.) Buijsen | LC | - | - | - | ✔️ | - | ✔️ | Rare-Sporadic | ✔️ | ✔️ | ✔️ | ✔️ |
| 179 | Fabaceae | *Koompassia malaccensis* Maing. ex Benth. | LR | - | ✔️ | - | - | - | ✔️ | Rare-Sporadic | ✔️ | ✔️ | ✔️ | - |
| 180 | Fabaceae | *Ormosia bancana* (Miq.) Merr. | - | - | - | - | - | - | - | Rare-Clustery | ✔️ | - | - | - |
| 181 | Fabaceae | *Ormosia macrodisca* Baker | LC | - | - | - | - | - | - | Rare-Clustery | ✔️ | - | - | - |
| 182 | Fabaceae | *Parkia speciosa* Hassk. | LC | - | - | ✔️ | ✔️ | ✔️ | ✔️ | Rare-Clustery | ✔️ | ✔️ | ✔️ | - |
| 183 | Fabaceae | *Parkia timoriana* (DC.) Merr | - | - | ✔️ | ✔️ | - | ✔️ | ✔️ | Rare-Clustery | ✔️ | ✔️ | ✔️ | - |
| 184 | Fabaceae | *Sindora leiocarpa* Backer ex K.Heyne & de Wit | LC | - | - | - | - | - | - | Rare-Clustery | ✔️ | - | - | - |
| 185 | Fabaceae | *Sindora velutina* Baker | LC | - | - | - | - | - | - | Rare-Clustery | ✔️ | - | - | - |
| 186 | Fabaceae | *Sindora wallichii* Benth. | LC | - | - | - | - | ✔️ | - | Rare-Clustery | ✔️ | - | - | - |
| 187 | Fagaceae | *Castanopsis fulva* Gamble | - | - | - | - | - | - | - | Rare-Clustery | ✔️ | - | - | - |
| 188 | Fagaceae | *Castanopsis motleyana* King | - | ✔️ | - | - | - | - | - | Rare-Clustery | ✔️ | - | - | - |
| 189 | Fagaceae | *Castanopsis tonkinensis* Seemen | - | - | - | - | - | - | - | Rare-Clustery | ✔️ | - | - | - |
| 190 | Fagaceae | *Lithocarpus gracilis* (Korth.) Soepadmo | LC | - | ✔️ | - | - | ✔️ | - | Rare-Widely spread | ✔️ | - | ✔️ | - |
| 191 | Fagaceae | *Lithocarpus bancanus* (Scheff.) Rehder | - | - | - | - | - | - | - | Rare-Clustery | ✔️ | - | - | - |
| 192 | Fagaceae | *Lithocarpus coopertus* (Blanco) Rehder | LC | - | - | - | - | ✔️ | - | Rare-Sporadic | ✔️ | - | - | - |
| 193 | Fagaceae | *Lithocarpus rassa* (Miq.) Rehder | - | - | ✔️ | - | - | - | - | Rare-Clustery | ✔️ | - | ✔️ | - |
| 194 | Fagaceae | *Quercus gemelliflora* Blume | LC | - | ✔️ | - | - | ✔️ | - | Rare-Clustery | ✔️ | - | ✔️ | - |
| 195 | Gentianaceae | *Fagraea renae* K.M.Wong & Sugau | - | - | - | - | - | - | - | Rare-Clustery | ✔️ | - | - | - |
| 196 | Hypericaceae | *Cratoxylum arborescens* (Vahl) Blume | LC | - | ✔️ | - | - | - | ✔️ | Rare-Clustery | ✔️ | ✔️ | ✔️ | - |
| 197 | Hypericaceae | *Cratoxylum sumatranum* (Jack) Blume | LC | - | - | - | - | - | ✔️ | Rare-Widely spread | ✔️ | ✔️ | - | - |
| 198 | Icacinaceae | *Stemonurus scorpioides* Becc. | - | - | - | - | - | - | ✔️ | Rare-Clustery | ✔️ | ✔️ | - | - |
| 199 | Irvingiaceae | *Irvingia malayana* Oliv. ex A.W.Benn. | LC | - | ✔️ | - | - | - | ✔️ | Rare-Clustery | ✔️ | ✔️ | ✔️ | - |
| 200 | Ixonanthaceae | *Ixonanthes petiolaris* Blume | - | - | - | - | - | - | - | Rare-Clustery | ✔️ | - | - | - |
| 201 | Ixonanthaceae | *Ixonanthes reticulata* Jack. | - | - | - | - | - | - | - | Rare-Clustery | ✔️ | - | - | - |
| 202 | Lamiaceae | *Callicarpa pentandra* Roxb. | LC | - | - | - | - | ✔️ | ✔️ | Rare-Sporadic | ✔️ | ✔️ | - | ✔️ |
| 203 | Lamiaceae | *Gmelina arborea* Roxb. ex Sm | LC | - | - | - | - | - | ✔️ | Rare-Clustery | ✔️ | ✔️ | - | - |
| 204 | Lamiaceae | *Peronema canescens* Jack. | LC | - | ✔️ | - | ✔️ | - | ✔️ | Rare-Clustery | ✔️ | ✔️ | ✔️ | - |
| 205 | Lamiaceae | *Vitex pinnata* L. | LC | - | - | - | ✔️ | - | ✔️ | Moderate-Widely spread | ✔️ | ✔️ | ✔️ | - |
| 206 | Lamiaceae | *Vitex trifolia* L. | - | - | - | - | ✔️ | - | - | Rare-Clustery | ✔️ | - | ✔️ | ✔️ |
| 207 | Lauraceae | *Actinodaphne glabra* Blume | LC | - | - | - | - | - | - | Rare-Widely spread | ✔️ | - | - | ✔️ |
| 208 | Lauraceae | *Alseodaphne elmeri* Merr. | Vu | ✔️ | - | - | - | ✔️ | - | Rare-Sporadic | ✔️ | - | - | ✔️ |
| 209 | Lauraceae | *Beilschmiedia dictyoneura* Kosterm. | VU | - | - | - | - | ✔️ | - | Rare-Clustery | ✔️ | - | - | - |
| 210 | Lauraceae | *Cryptocarya diversifolia*Blume | - | - | - | - | - | ✔️ | - | Rare-Clustery | ✔️ | - | - | - |
| 211 | Lauraceae | *Cryptocarya impressa* Miq. | LC | - | - | - | - | ✔️ | - | Rare-Clustery | ✔️ | - | - | - |
| 212 | Lauraceae | *Endiandra coriacea* Merr. | - | - | - | - | - | - | - | Rare-Clustery | ✔️ | - | - | - |
| 213 | Lauraceae | *Endiandra kingiana* Gamble | LC | - | - | - | - | - | ✔️ | Rare-Sporadic | ✔️ | ✔️ | - | - |
| 214 | Lauraceae | *Eusideroxylon zwageri* Teijsm.&Binn. | Vu | - | ✔️ | - | ✔️ | ✔️ | - | Rare-Widely spread | ✔️ | - | ✔️ | ✔️ |
| 215 | Lauraceae | *Litsea angulata* Blume | - | - | - | - | ✔️ | ✔️ | ✔️ | Rare-Sporadic | ✔️ | ✔️ | ✔️ | - |
| 216 | Lauraceae | *Litsea elliptica* Blume | LC | - | ✔️ | - | - | ✔️ | ✔️ | Rare-Sporadic | ✔️ | ✔️ | ✔️ | - |
| 217 | Lauraceae | *Litsea garciae* S.Vidal | LC | - | - | ✔️ | ✔️ | ✔️ | - | Rare-Sporadic | ✔️ | - | ✔️ | - |
| 218 | Lauraceae | *Litsea oppositifolia* L.S. Gibbs | - | - | - | - | - | ✔️ | - | Rare-Clustery | ✔️ | - | - | - |
| 219 | Lauraceae | *Litsea umbellata* (Lour.) Merr. | LC | - | ✔️ | - | ✔️ | ✔️ | - | Rare-Sporadic | ✔️ | - | ✔️ | - |
| 220 | Lauraceae | *Neolitsea latifolia* (Blume) S.Moore | - | - | - | - | - | - | - | Rare-Clustery | ✔️ | - | - | ✔️ |
| 221 | Lecythidaceae | *Barringtonia macrostachya* (Jack) Kurz | - | - | - | - | - | ✔️ | - | Rare-Widely spread | ✔️ | - | - | ✔️ |
| 222 | Lecythidaceae | *Barringtonia pendula* (Griff.) Kurz | - | - | - | - | - | ✔️ | - | Rare-Clustery | ✔️ | - | - | - |
| 223 | Lecythidaceae | *Barringtonia reticulata* (Blume) Miq. | LC | - | - | - | - | ✔️ | - | Rare-Clustery | ✔️ | - | - | ✔️ |
| 224 | Lecythidaceae | *Planchonia valida* (Blume) Blume | - | - | ✔️ | - | - | - | - | Rare-Clustery | ✔️ | - | ✔️ | - |
| 225 | Lythraceae | *Duabanga moluccana* Blume | LC | - | - | - | - | - | ✔️ | Rare-Clustery | ✔️ | ✔️ | - | - |
| 226 | Lythraceae | *Lagerstroemia speciosa* (L.) Pers. | - | - | ✔️ | - | ✔️ | - | ✔️ | Rare-Clustery | ✔️ | ✔️ | ✔️ | ✔️ |
| 227 | Magnoliaceae | *Magnolia lasia* Noot. | DD | ✔️ | - | - | - | - | - | Rare-Clustery | ✔️ | - | - | - |
| 228 | Magnoliaceae | *Magnolia tsiampacca* (L.) Figlar & Noot. | DD | - | ✔️ | - | - | - | - | Rare-Clustery | ✔️ | - | ✔️ | ✔️ |
| 229 | Malvaceae | *Boschia griffithii* Mast. | VU | - | - | - | - | - | ✔️ | Rare-Sporadic | ✔️ | ✔️ | - | ✔️ |
| 230 | Malvaceae | *Brownlowia peltata* Benth. | - | ✔️ | - | - | - | - | - | Rare-Clustery | ✔️ | - | - | - |
| 231 | Malvaceae | *Durio dulcis* Becc. | Vu | ✔️ | - | ✔️ | - | ✔️ | - | Rare-Clustery | ✔️ | - | ✔️ | - |
| 232 | Malvaceae | *Durio graveolens* Becc. | Vu | - | - | ✔️ | - | ✔️ | - | Rare-Clustery | ✔️ | - | ✔️ | - |
| 233 | Malvaceae | *Durio kutejensis* (Hassk.) Becc. | Vu | ✔️ | - | ✔️ | ✔️ | ✔️ | - | Abundance-Clustery | ✔️ | - | ✔️ | - |
| 234 | Malvaceae | *Durio lanceolatus* Mast. | NT | ✔️ | ✔️ | - | - | ✔️ | - | Rare-Clustery | ✔️ | - | ✔️ | - |
| 235 | Malvaceae | *Durio oxleyanus* Griff. | Vu | - | - | ✔️ | - | ✔️ | - | Rare-Sporadic | ✔️ | - | ✔️ | - |
| 236 | Malvaceae | *Durio zibethinus* L. | - | - | ✔️ | ✔️ | ✔️ | ✔️ | - | Abundance-Clustery | ✔️ | - | ✔️ | - |
| 237 | Malvaceae | *Heritiera elata* Ridl. | - | - | - | - | - | - | ✔️ | Rare-Sporadic | ✔️ | ✔️ | - | - |
| 238 | Malvaceae | *Heritiera simplicifolia* (Mast.) Kosterm. | - | - | ✔️ | - | - | - | ✔️ | Rare-Clustery | ✔️ | ✔️ | ✔️ | - |
| 239 | Malvaceae | *Heritiera sumatrana* (Miq.) Kosterm. | - | - | - | - | - | - | - | Rare-Clustery | ✔️ | - | - | - |
| 240 | Malvaceae | *Neesia synandra* Mast. | - | - | ✔️ | - | - | ✔️ | - | Rare-Clustery | ✔️ | - | ✔️ | - |
| 241 | Malvaceae | *Pentace erectinervia* Kosterm. | - | ✔️ | ✔️ | - | - | - | ✔️ | Rare-Clustery | ✔️ | ✔️ | ✔️ | - |
| 242 | Malvaceae | *Pentace laxiflora* Merr. | LC | ✔️ | ✔️ | - | - | - | ✔️ | Rare-Widely spread | ✔️ | ✔️ | ✔️ | - |
| 243 | Malvaceae | *Pentace triptera* Mast. | LC | - | ✔️ | - | - | - | ✔️ | Rare-Sporadic | ✔️ | ✔️ | ✔️ | - |
| 244 | Malvaceae | *Pterospermum diversifolium* Blume | LC | - | ✔️ | ✔️ | - | ✔️ | - | Rare-Clustery | ✔️ | - | ✔️ | - |
| 245 | Malvaceae | *Pterospermum javanicum* Jungh. | LC | - | ✔️ | ✔️ | ✔️ | ✔️ | ✔️ | Rare-Clustery | ✔️ | ✔️ | ✔️ | - |
| 246 | Malvaceae | *Microcos crassifolia* Burret. | - | - | - | ✔️ | - | ✔️ | ✔️ | Rare-Sporadic | ✔️ | ✔️ | ✔️ | - |
| 247 | Malvaceae | *Microcos tomentosa* Sm. | LC | - | - | - | - | ✔️ | ✔️ | Rare-Sporadic | ✔️ | ✔️ | - | - |
| 248 | Malvaceae | *Scaphium macropodum* (Miq.) Beumée ex K.Heyne | LC | - | - | - | - | - | ✔️ | Rare-Sporadic | ✔️ | ✔️ | - | - |
| 249 | Malvaceae | *Sterculia oblongata*R.Br. | - | - | - | - | - | - | - | Rare-Clustery | ✔️ | - | - | - |
| 250 | Malvaceae | *Sterculia rubiginosa* Vent. | - | - | - | - | - | - | ✔️ | Rare-Sporadic | ✔️ | ✔️ | - | - |
| 251 | Malvaceae | *Boschia excelsa* Korth. | LC | - | - | - | - | - | - | Rare-Clustery | ✔️ | - | - | - |
| 252 | Melastomataceae | *Pternandra azurea* (DC.) Burkill | - | - | - | - | - | - | ✔️ | Rare-Clustery | ✔️ | ✔️ | - | - |
| 253 | Melastomataceae | *Pternandra coerulescens* Jack | - | - | - | - | - | - | ✔️ | Rare-Clustery | ✔️ | ✔️ | - | - |
| 254 | Melastomataceae | *Pternandra galeata* Ridl. | - | - | - | - | ✔️ | ✔️ | ✔️ | Moderate-Widely spread | ✔️ | ✔️ | ✔️ | - |
| 255 | Melastomataceae | *Pternandra rostrata* (Cogn.) M.P.Nayar | - | - | - | - | ✔️ | ✔️ | ✔️ | Rare-Widely spread | ✔️ | ✔️ | ✔️ | - |
| 256 | Melastomataceae | *Memecylon lilacinum* Zoll. & Moritzi | - | - | - | - | - | - | ✔️ | Rare-Clustery | ✔️ | ✔️ | - | - |
| 257 | Melastomataceae | *Memecylon edule* Roxb. | - | - | - | - | - | - | - | Rare-Clustery | ✔️ | - | - | - |
| 258 | Melastomataceae | *Memecylon minutiflorum* Miq. | - | - | - | - | - | - | - | Rare-Clustery | ✔️ | - | - | - |
| 259 | Meliaceae | *Aglaia argentea* Blume | LC | - | - | - | - | - | - | Rare-Clustery | ✔️ | - | - | - |
| 260 | Meliaceae | *Aglaia crassinervia* Kurz ex Hiern | NT | - | - | - | - | ✔️ | - | Rare-Clustery | ✔️ | - | - | - |
| 261 | Meliaceae | *Aglaia korthalsii* Miq. | NT | - | - | - | - | ✔️ | - | Rare-Clustery | ✔️ | - | - | - |
| 262 | Meliaceae | *Aglaia simplicifolia* (Bedd.) Harms | NT | - | - | - | - | ✔️ | ✔️ | Rare-Clustery | ✔️ | ✔️ | - | - |
| 263 | Meliaceae | *Aglaia tomentosa* Teijsm. & Binn. | LC | - | - | - | - | ✔️ | ✔️ | Rare-Clustery | ✔️ | ✔️ | - | - |
| 264 | Meliaceae | *Heynea trijuga* Roxb. ex Sims | LC | - | - | - | ✔️ | - | ✔️ | Rare-Clustery | ✔️ | ✔️ | ✔️ | - |
| 265 | Meliaceae | *Lansium domesticum* Corrêa | - | - | - | ✔️ | ✔️ | ✔️ | - | Moderate-Clustery | ✔️ | - | ✔️ | - |
| 266 | Meliaceae | *Sandoricum borneense* Miq. | - | ✔️ | - | ✔️ | - | ✔️ | - | Rare-Clustery | ✔️ | - | ✔️ | - |
| 267 | Meliaceae | *Sandoricum koetjape* (Burm.f.) Merr. | LC | - | - | ✔️ | ✔️ | ✔️ | - | Rare-Clustery | ✔️ | - | ✔️ | - |
| 268 | Moraceae | *Artocarpus anisophyllus* Miq. | VU | - | - | ✔️ | ✔️ | ✔️ | ✔️ | Rare-Widely spread | ✔️ | ✔️ | ✔️ | - |
| 269 | Moraceae | *Artocarpus elasticus* Reinw. ex Blume | LC | - | - | ✔️ | - | ✔️ | ✔️ | Rare-Widely spread | ✔️ | ✔️ | ✔️ | - |
| 270 | Moraceae | *Artocarpus dadah* Miq. | - | - | - | ✔️ | - | ✔️ | ✔️ | Rare-Clustery | ✔️ | ✔️ | ✔️ | - |
| 271 | Moraceae | *Artocarpus fretessii* Teijsm. & Binn. ex Hassk. | - | - | - | - | - | - | - | Rare-Clustery | ✔️ | - | - | - |
| 272 | Moraceae | *Artocarpus heterophyllus* Lam. | - | - | - | ✔️ | ✔️ | ✔️ | ✔️ | Rare-Clustery | ✔️ | ✔️ | ✔️ | - |
| 273 | Moraceae | *Artocarpus integer* (Thunb.) Merr. | - | - | - | ✔️ | ✔️ | ✔️ | ✔️ | Moderate-Sporadic | ✔️ | ✔️ | ✔️ | - |
| 274 | Moraceae | *Artocarpus kemando* Miq. | - | - | - | - | - | ✔️ | - | Rare-Sporadic | ✔️ | - | - | ✔️ |
| 275 | Moraceae | *Artocarpus lamellosus*Blanco | LC | - | - | ✔️ | - | ✔️ | ✔️ | Rare-Sporadic | ✔️ | ✔️ | ✔️ | - |
| 276 | Moraceae | *Artocarpus lanceifolius* Roxb. | - | - | - | ✔️ | ✔️ | ✔️ | ✔️ | Rare-Sporadic | ✔️ | ✔️ | ✔️ | - |
| 277 | Moraceae | *Artocarpus odoratissimus* Blanco | NT | - | - | ✔️ | ✔️ | ✔️ | ✔️ | Rare-Clustery | ✔️ | ✔️ | ✔️ | ✔️ |
| 278 | Moraceae | *Artocarpus rigidus* Blume | - | - | - | ✔️ | ✔️ | ✔️ | ✔️ | Rare-Widely spread | ✔️ | ✔️ | ✔️ | ✔️ |
| 279 | Moraceae | *Artocarpus tamaran* Becc. | VU | ✔️ | - | ✔️ | - | ✔️ | - | Rare-Sporadic | ✔️ | - | ✔️ | - |
| 280 | Moraceae | *Ficus bukitrayaensis* C.C.Berg | LC | ✔️ | - | - | - | ✔️ | - | Rare-Clustery | ✔️ | - | - | ✔️ |
| 281 | Moraceae | *Ficus fistulosa* Reinw. ex Blume | LC | - | - | ✔️ | ✔️ | ✔️ | - | Rare-Clustery | ✔️ | - | ✔️ | - |
| 282 | Moraceae | *Ficus* *variegata* Blume | - | - | ✔️ | ✔️ | ✔️ | ✔️ | ✔️ | Rare-Widely spread | ✔️ | ✔️ | ✔️ | ✔️ |
| 283 | Moraceae | *Parartocarpus bracteatus* (King) Becc. | - | - | - | - | - | ✔️ | ✔️ | Rare-Clustery | ✔️ | ✔️ | - | - |
| 284 | Myristicaceae | *Horsfieldia gracilis* W.J.de Wilde | VU | ✔️ | - | - | - | - | - | Rare-Clustery | ✔️ | - | - | - |
| 285 | Myristicaceae | *Horsfieldia irya* (Gaertn.) Warb. | LC | - | - | - | - | ✔️ | - | Rare-Clustery | ✔️ | - | - | - |
| 286 | Myristicaceae | *Horsfieldia reticulata* Warb. | NT | ✔️ | - | ✔️ | - | ✔️ | - | Rare-Clustery | ✔️ | - | ✔️ | - |
| 287 | Myristicaceae | *Gymnacranthera bancana* (Miq.) J.Sinclair | - | - | - | - | - | - | - | Rare-Clustery | ✔️ | - | - | - |
| 288 | Myristicaceae | *Gymnacranthera farquhariana* (Hokk.&Thomson) Warb. | - | - | ✔️ | - | - | ✔️ | ✔️ | Rare-Sporadic | ✔️ | ✔️ | ✔️ | - |
| 289 | Myristicaceae | *Gymnacranthera forbesii* (King) Warb. | - | - | - | - | - | ✔️ | - | Rare-Clustery | ✔️ | - | - | - |
| 290 | Myristicaceae | *Horsfieldia polyspherula* (Hook.f. & emend. King) J.Sinclair | VU | - | - | - | - | ✔️ | - | Rare-Sporadic | ✔️ | - | - | - |
| 291 | Myristicaceae | *Knema glauca* (Blume (Warb) | - | - | - | - | - | ✔️ | - | Rare-Clustery | ✔️ | - | - | - |
| 292 | Myristicaceae | *Knema kunstleri* (King) Warb. | - | - | - | - | - | - | - | Rare-Clustery | ✔️ | - | - | - |
| 293 | Myristicaceae | *Knema latericia* Elmer | - | - | - | - | - | ✔️ | - | Rare-Clustery | ✔️ | - | - | ✔️ |
| 294 | Myristicaceae | *Knema latifolia* Warb. | LC | - | - | - | - | ✔️ | - | Rare-Sporadic | ✔️ | - | - | - |
| 295 | Myristicaceae | *Knema laurina* Warb. | LC | - | - | - | - | ✔️ | - | Rare-Clustery | ✔️ | - | - | - |
| 296 | Myristicaceae | *Knema membranifolia* H.J.P. Winkl. | - | ✔️ | - | - | - | ✔️ | - | Rare-Clustery | ✔️ | - | - | - |
| 297 | Myristicaceae | *Knema pallens* W.J.de Wilde | - | ✔️ | - | - | - | ✔️ | - | Rare-Sporadic | ✔️ | - | - | - |
| 298 | Myristicaceae | *Knema percoriacea* J.Sinclair | - | ✔️ | - | - | - | ✔️ | - | Rare-Sporadic | ✔️ | - | - | - |
| 299 | Myristicaceae | *Knema psilantha* W.J.J.O.de Wilde | - | ✔️ | - | - | - | ✔️ | - | Rare-Clustery | ✔️ | - | - | - |
| 300 | Myristicaceae | *Myristica maxima* Warb. | LC | - | - | - | - | ✔️ | - | Rare-Clustery | ✔️ | - | - | - |
| 301 | Myristicaceae | *Myristica simiarum* A. DC. | VU | - | ✔️ | - | - | ✔️ | - | Rare-Clustery | ✔️ | - | ✔️ | - |
| 302 | Myristicaceae | *Myristica villosa* Warb. | - | ✔️ | - | - | - | ✔️ | - | Rare-Sporadic | ✔️ | - | - | - |
| 303 | Myrtaceae | *Rhodamnia cinerea* Jack | LC | - | - | - | ✔️ | - | ✔️ | Rare-Sporadic | ✔️ | ✔️ | ✔️ | ✔️ |
| 304 | Myrtaceae | *Syzygium cerasiforme* (Blume) Merr. & L.M.Perry | - | - | - | - | - | - | ✔️ | Rare-Clustery | ✔️ | ✔️ | - | - |
| 305 | Myrtaceae | *Syzygium dyerianum* (King) Chantar. & J.Parn. | LC | - | - | - | - | - | ✔️ | Rare-Clustery | ✔️ | ✔️ | - | - |
| 306 | Myrtaceae | *Syzygium koordersianum* (King) I.M.Turner | - | - | - | - | - | - | ✔️ | Rare-Clustery | ✔️ | ✔️ | - | - |
| 307 | Myrtaceae | *Syzygium linocieroideum* (King) I.M.Turner | NT | - | - | - | - | - | ✔️ | Rare-Clustery | ✔️ | ✔️ | - | - |
| 308 | Myrtaceae | *Syzygium malaccense* (L.) Merr. & L.M.Perry | LC | - | - | ✔️ | ✔️ | ✔️ | - | Rare-Clustery | ✔️ | - | ✔️ | - |
| 309 | Myrtaceae | *Syzygium napiforme* (Koord. & Valeton) Merr. & L.M.Perry | - | - | - | - | - | - | ✔️ | Rare-Clustery | ✔️ | ✔️ | - | - |
| 310 | Myrtaceae | *Syzygium nigricans* (King) Merr. & L.M.Perry | - | - | - | - | - | - | ✔️ | Rare-Clustery | ✔️ | ✔️ | - | ✔️ |
| 311 | Myrtaceae | *Syzygium scortechinii* (King) Chantar. & J.Parn. | EN | - | - | - | - | - | - | Rare-Clustery | ✔️ | - | - | - |
| 312 | Myrtaceae | *Syzygium stapfianum* (King) I.M.Turner | - | - | - | - | - | ✔️ | - | Rare-Clustery | ✔️ | - | - | - |
| 313 | Myrtaceae | *Syzygium tawahense* (Korth.) Merr. & Perry | LC | ✔️ | - | - | - | ✔️ | ✔️ | Rare-Sporadic | ✔️ | ✔️ | - | - |
| 314 | Myrtaceae | *Syzygium zeylanicum* (L.) DC. | - | - | - | - | - | - | ✔️ | Rare-Clustery | ✔️ | ✔️ | - | ✔️ |
| 315 | Ochnaceae | *Brackenridgea palustris* Bartell. | NT | - | - | - | - | - | - | Rare-Clustery | ✔️ | - | - | - |
| 316 | Ochnaceae | *Campylospermum serratum* (Gaertn.) Bittrich & M.C.E.Amaral | LC | - | - | - | - | - | - | Rare-Clustery | ✔️ | - | - | - |
| 317 | Olacaceae | *Ochanostachys amentacea* Mast. | DD | - | ✔️ | - | - | - | ✔️ | Rare-Sporadic | ✔️ | ✔️ | ✔️ | - |
| 318 | Olacaceae | *Scorodocarpus borneensis* (Baill.) Becc. | - | - | ✔️ | ✔️ | ✔️ | ✔️ | - | Rare-Sporadic | ✔️ | - | ✔️ | - |
| 319 | Olacaceae | *Strombosia ceylanica* Gardner | - | - | - | - | - | - | - | Rare-Clustery | ✔️ | - | - | - |
| 320 | Oxalidaceae | *Sarcotheca diversifolia* (Miq.) Hallier f. | - | - | - | - | - | - | - | Rare-Clustery | ✔️ | - | ✔️ | ✔️ |
| 321 | Olacaceae | *Strombosia javanica* Blume | - | - | - | ✔️ | - | ✔️ | - | Rare-Sporadic | ✔️ | - | ✔️ | - |
| 322 | Pentaphylacaceae | *Adinandra collina* Kobuski | - | ✔️ | - | - | - | - | - | Rare-Clustery | ✔️ | - | - | - |
| 323 | Pentaphylacaceae | *Adinandra dumosa* Jack | LC | - | - | - | - | ✔️ | ✔️ | Rare-Clustery | ✔️ | ✔️ | - | - |
| 324 | Pentaphylacaceae | *Adinandra subsessilis* Airy Shaw | - | ✔️ | - | - | - | - | ✔️ | Rare-Clustery | ✔️ | ✔️ | - | - |
| 325 | Peraceae | *Chaetocarpus castanicarpus*(Roxb.) Thwaites | LC | - | - | - | - | ✔️ | - | Rare-Sporadic | ✔️ | - | - | - |
| 326 | Phyllanthaceae | *Aporosa falcifera* Hook.f. | - | - | - | - | - | - | - | Rare-Clustery | ✔️ | - | - | - |
| 327 | Phyllanthaceae | *Aporosa grandistipula* Merr. | - | - | - | - | - | - | - | Rare-Clustery | ✔️ | - | - | - |
| 328 | Phyllanthaceae | *Aporosa lucida* (Miq.) Airy Shaw | - | - | - | - | - | - | ✔️ | Rare-Clustery | ✔️ | ✔️ | - | - |
| 329 | Phyllanthaceae | *Aporosa nitida* Merr. | - | ✔️ | - | - | - | ✔️ | ✔️ | Rare-Widely spread | ✔️ | ✔️ | - | - |
| 330 | Phyllanthaceae | *Aporosa octandra* (Buch.-Ham. ex D.Don) Vickery | LC | - | - | - | - | - | - | Rare-Clustery | ✔️ | - | - | - |
| 331 | Phyllanthaceae | *Aporosa subcaudata* Merr. | - | - | - | - | - | - | ✔️ | Rare-Sporadic | ✔️ | ✔️ | - | - |
| 332 | Phyllanthaceae | *Antidesma coriaceum* Tul. | - | - | - | - | - | - | - | Rare-Clustery | ✔️ | - | - | - |
| 333 | Phyllanthaceae | *Baccaurea bracteata* Müll.Arg. | - | - | - | ✔️ | - | ✔️ | - | Rare-Sporadic | ✔️ | - | ✔️ | ✔️ |
| 334 | Phyllanthaceae | *Baccaurea glaucescens* (Chodat & Hassl.) Soria & Zardini | - | - | - | - | - | ✔️ | - | Rare-Clustery | ✔️ | - | - | ✔️ |
| 335 | Phyllanthaceae | *Baccaurea macrocarpa* (Miq.) Müll.Arg. | - | - | - | ✔️ | - | ✔️ | - | Rare-Clustery | ✔️ | - | ✔️ | ✔️ |
| 336 | Phyllanthaceae | *Baccaurea macrophylla* (Müll.Arg.) Müll.Arg. | - | - | - | - | - | ✔️ | - | Rare-Clustery | ✔️ | - | - | ✔️ |
| 337 | Phyllanthaceae | *Baccaurea motleyana* (Müll.Arg.) Müll.Arg. | LC | - | - | ✔️ | ✔️ | ✔️ | - | Rare-Clustery | ✔️ | - | ✔️ | ✔️ |
| 338 | Phyllanthaceae | *Baccaurea odoratissima* Elmer | VU | - | - | - | - | ✔️ | - | Rare-Sporadic | ✔️ | - | - | ✔️ |
| 339 | Phyllanthaceae | *Baccaurea polyneura* Hook.f. | LR | - | - | - | - | - | - | Rare-Clustery | ✔️ | - | - | ✔️ |
| 340 | Phyllanthaceae | *Baccaurea pyriformis* Gage | LC | - | - | ✔️ | - | ✔️ | - | Rare-Clustery | ✔️ | - | ✔️ | ✔️ |
| 341 | Phyllanthaceae | *Baccaurea sumatrana* (Miq.) Mull.Arg. | - | - | - | ✔️ | - | ✔️ | ✔️ | Rare-Clustery | ✔️ | ✔️ | ✔️ | ✔️ |
| 342 | Phyllanthaceae | *Baccaurea tetrandra* (Baill.) Müll.Arg. | LC | - | - | ✔️ | - | ✔️ | - | Rare-Clustery | ✔️ | - | ✔️ | ✔️ |
| 343 | Phyllanthaceae | *Bridelia glauca* Blume | LC | - | - | - | ✔️ | - | ✔️ | Rare-Sporadic | ✔️ | ✔️ | ✔️ | ✔️ |
| 344 | Phyllanthaceae | *Glochidion obscurum* (Roxb.ex Willd.) Blume | - | - | - | - | - | ✔️ | ✔️ | Rare-Clustery | ✔️ | ✔️ | - | - |
| 345 | Phyllanthaceae | *Glochidion sericeum* (Blume) Zoll. & Moritzi | - | - | - | - | - | ✔️ | ✔️ | Rare-Sporadic | ✔️ | ✔️ | - | - |
| 346 | Phyllanthaceae | *Cleistanthus erycibifolius* Airy Shaw | - | - | - | - | - | - | - | Rare-Clustery | ✔️ | - | - | - |
| 347 | Phyllanthaceae | *Cleistanthus oblongifolius* (Roxb.) Müll.Arg. | LC | - | - | - | - | - | - | Rare-Clustery | ✔️ | - | - | - |
| 348 | Podocarpaceae | *Nageia wallichiana* (C.Presl) Kuntze | LC | - | - | - | - | - | - | Rare-Clustery | ✔️ | - | - | ✔️ |
| 349 | Polygalaceae | *Xanthophyllum amoenum* Chodat | - | - | - | - | - | ✔️ | - | Rare-Clustery | ✔️ | - | - | - |
| 350 | Polygalaceae | *Xanthophyllum discolor* Chodat | - | - | - | - | - | - | - | Rare-Clustery | ✔️ | - | - | - |
| 351 | Polygalaceae | *Xanthophyllum flavescens* Roxb. | - | - | ✔️ | - | - | - | - | Rare-Clustery | ✔️ | - | ✔️ | - |
| 352 | Polygalaceae | *Xanthophyllum obscurum* A.W.Benn. | - | - | - | ✔️ | - | ✔️ | ✔️ | Rare-Clustery | ✔️ | ✔️ | ✔️ | - |
| 353 | Polygalaceae | *Xanthophyllum rufum* A.W.Benn. | - | ✔️ | - | - | - | - | - | Rare-Clustery | ✔️ | - | - | - |
| 354 | Polygalaceae | *Xanthophyllum stipitatum* A.W.Benn. | - | - | - | ✔️ | - | ✔️ | ✔️ | Rare-Clustery | ✔️ | ✔️ | ✔️ | - |
| 355 | Putranjivaceae | *Drypetes longifolia* (Blume) Pax & K.Hoffm. | LC | - | - | - | - | - | - | Rare-Clustery | ✔️ | - | - | - |
| 356 | Putranjivaceae | *Drypetes kikir* Airy Shaw. | - | - | - | - | - | - | ✔️ | Rare-Clustery | ✔️ | ✔️ | - | - |
| 357 | Putranjivaceae | *Drypetes oblongifolia* (Bedd.) Airy Shaw | - | - | - | - | - | - | - | Rare-Sporadic | ✔️ | - | - | - |
| 358 | Putranjivaceae | *Drypetes polyneura* Airy Shaw | - | - | ✔️ | - | - | - | - | Rare-Clustery | ✔️ | - | ✔️ | - |
| 359 | Rhamnaceae | *Ziziphus angustifolia*(Miq.) Hatus. ex Steenis | - | - | - | - | - | - | ✔️ | Rare-Clustery | ✔️ | ✔️ | - | - |
| 360 | Rhizophoraceae | *Carallia brachiata* (Lour.) Merr. | - | - | - | - | - | - | ✔️ | Rare-Clustery | ✔️ | ✔️ | - | - |
| 361 | Rhizophoraceae | *Pellacalyx axillaris* Korth. | - | - | - | - | - | - | ✔️ | Rare-Clustery | ✔️ | ✔️ | - | - |
| 362 | Rosaceae | *Prunus arborea* (Blume) Kalkman | LC | - | - | - | - | ✔️ | ✔️ | Rare-Clustery | ✔️ | ✔️ | - | - |
| 363 | Rosaceae | *Prunus beccarii* (Ridl.) Kalkman | - | ✔️ | - | - | - | - | - | Rare-Sporadic | ✔️ | - | - | - |
| 364 | Rosaceae | *Prunus javanica* (Teijsm. & Binn.) Miq. | LC | - | - | - | - | ✔️ | - | Rare-Clustery | ✔️ | - | - | - |
| 365 | Rubiaceae | *Adina eurhyncha* (Miq.) Å.Krüger & Löfstrand | - | - | - | - | ✔️ | - | ✔️ | Rare-Sporadic | ✔️ | ✔️ | ✔️ | - |
| 366 | Rubiaceae | *Aidia densiflora* (Benth.) Masamune | - | - | - | - | ✔️ | - | ✔️ | Rare-Clustery | ✔️ | ✔️ | ✔️ | - |
| 367 | Rubiaceae | *Dibridsonia conferta* (Korth.) K.M.Wong | - | - | - | - | - | - | - | Rare-Clustery | ✔️ | - | - | - |
| 368 | Rubiaceae | *Gardenia tubifera* Wall. ex Roxb. | - | - | - | - | - | - | ✔️ | Rare-Clustery | ✔️ | ✔️ | - | - |
| 369 | Rubiaceae | *Jackiopsis ornata* (Wall.) Ridsdale | - | - | - | - | - | - | - | Rare-Clustery | ✔️ | - | - | - |
| 370 | Rubiaceae | *Nauclea officinalis* (Pierre ex Pit.) Merr. & Chun | - | - | - | - | - | - | ✔️ | Rare-Widely spread | ✔️ | ✔️ | - | - |
| 371 | Rubiaceae | *Nauclea subdita* (Korth.) Steud. | LC | - | - | - | - | - | ✔️ | Rare-Sporadic | ✔️ | ✔️ | - | - |
| 372 | Rubiaceae | *Neolamarckia cadamba* (Roxb.) Bosser | - | - | - | - | ✔️ | - | - | Rare-Sporadic | ✔️ | - | ✔️ | - |
| 373 | Rubiaceae | *Neonauclea gigantea* (Valeton) Merr. | - | - | - | - | ✔️ | - | ✔️ | Rare-Sporadic | ✔️ | ✔️ | ✔️ | - |
| 374 | Rubiaceae | *Ochreinauclea maingayi* (Hook.f.) Ridsdale | LC | - | - | - | - | - | - | Rare-Clustery | ✔️ | - | - | - |
| 375 | Rubiaceae | *Porterandia anisophylla* (Jack ex Roxb.) Ridl. | - | - | - | - | - | - | ✔️ | Rare-Clustery | ✔️ | ✔️ | - | - |
| 376 | Rubiaceae | *Ridsdalea grandis* (Korth.) J.T.Pereira | - | - | - | - | - | - | - | Rare-Clustery | ✔️ | - | - | ✔️ |
| 377 | Rubiaceae | *Ridsdalea schoemannii*(Teijsm. & Binn.) J.T.Pereira | LC | - | - | - | - | - | - | Rare-Clustery | ✔️ | - | - | ✔️ |
| 378 | Rubiaceae | *Timonius flavescens* (Jack.) Baker | LC | - | - | - | ✔️ | - | ✔️ | Rare-Sporadic | ✔️ | ✔️ | ✔️ | - |
| 379 | Rubiaceae | *Urophyllum polyneurum* Miq. | - | - | - | - | - | - | ✔️ | Rare-Clustery | ✔️ | ✔️ | - | - |
| 380 | Rutaceae | *Citrus hystrix* DC. | - | - | - | ✔️ | ✔️ | ✔️ | - | Rare-Clustery | ✔️ | - | ✔️ | ✔️ |
| 381 | Rutaceae | *Melicope frutescens*(Blanco) Appelhans & J.Wen | LC | - | - | - | ✔️ | - | ✔️ | Rare-Sporadic | ✔️ | ✔️ | ✔️ | - |
| 382 | Sapindaceae | *Dimocarpus longan* Lour. | NT | - | - | ✔️ | ✔️ | ✔️ | ✔️ | Rare-Sporadic | ✔️ | ✔️ | ✔️ | - |
| 383 | Sapindaceae | *Guioa pleuropteris* (Blume) Radlk. | - | - | - | - | ✔️ | - | ✔️ | Rare-Clustery | ✔️ | ✔️ | ✔️ | - |
| 384 | Sapindaceae | *Mischocarpus sundaicus* Blume | - | - | - | - | - | - | - | Rare-Clustery | ✔️ | - | - | ✔️ |
| 385 | Sapindaceae | *Nephelium cuspidatum* Blume | LC | - | - | ✔️ | - | ✔️ | - | Rare-Clustery | ✔️ | - | ✔️ | ✔️ |
| 386 | Sapindaceae | *Nephelium lappaceum* L. | LC | - | - | ✔️ | ✔️ | ✔️ | ✔️ | Moderate-Sporadic | ✔️ | ✔️ | ✔️ | - |
| 387 | Sapindaceae | *Nephelium maingayi* Hiern | LC | - | - | ✔️ | - | ✔️ | ✔️ | Rare-Clustery | ✔️ | ✔️ | ✔️ | - |
| 388 | Sapindaceae | *Nephelium ramboutan-ake* (Labill.) Leenh. | - | - | - | ✔️ | ✔️ | ✔️ | - | Rare-Clustery | ✔️ | - | ✔️ | - |
| 389 | Sapindaceae | *Nephelium uncinatum* Radlk. ex Leenh. | LC | - | - | ✔️ | - | ✔️ | ✔️ | Rare-Clustery | ✔️ | ✔️ | ✔️ | ✔️ |
| 390 | Sapindaceae | *Pometia pinnata* J.R Forst. & G.R Forst. | LC | - | ✔️ | ✔️ | ✔️ | ✔️ | ✔️ | Rare-Sporadic | ✔️ | ✔️ | ✔️ | - |
| 391 | Sapindaceae | *Xerospermum noronhianum* (Blume) Blume | - | - | - | - | - | ✔️ | - | Rare-Clustery | ✔️ | - | - | - |
| 392 | Sapotaceae | *Madhuca kingiana* (Brace ex King & Gamble) H.J.Lam | NT | - | ✔️ | - | - | ✔️ | - | Moderate-Widely spread | ✔️ | - | ✔️ | - |
| 393 | Sapotaceae | *Madhuca longifolia* (J.Koenig ex L.) J.F.Macbr. | - | - | - | - | - | - | - | Rare-Clustery | ✔️ | - | - | - |
| 394 | Sapotaceae | *Madhuca motleyana* (de Vriese) J.F.Macbr. | NT | - | ✔️ | ✔️ | - | ✔️ | - | Rare-Clustery | ✔️ | - | ✔️ | - |
| 395 | Sapotaceae | *Madhuca pallida* (Burck) Baehni | NT | - | ✔️ | - | - | - | - | Rare-Clustery | ✔️ | - | ✔️ | - |
| 396 | Sapotaceae | *Madhuca pierrei*(F.N.Williams) H.J.Lam | - | - | - | - | - | - | - | Rare-Clustery | ✔️ | - | - | - |
| 397 | Sapotaceae | *Madhuca sericea* (Miq.) H.J.Lam | VU | - | - | - | - | - | ✔️ | Rare-Sporadic | ✔️ | ✔️ | - | - |
| 398 | Sapotaceae | *Palaquium beccarianum* (Pierre) Royen | LC | ✔️ | - | - | - | ✔️ | - | Rare-Clustery | ✔️ | - | - | - |
| 399 | Sapotaceae | *Palaquium dasyphyllum* Pierre ex Dubard | LC | ✔️ | - | - | - | - | - | Rare-Clustery | ✔️ | - | - | - |
| 400 | Sapotaceae | *Palaquium quercifolium* (de Vriese) Burck | LC | - | ✔️ | - | - | ✔️ | - | Rare-Sporadic | ✔️ | - | ✔️ | - |
| 401 | Sapotaceae | *Palaquium rostratum* (Miq.) Burck | LC | - | ✔️ | - | - | - | - | Rare-Clustery | ✔️ | - | ✔️ | - |
| 402 | Sapotaceae | *Palaquium sericeum* H.J.Lam | LC | ✔️ | ✔️ | - | - | ✔️ | ✔️ | Rare-Clustery | ✔️ | ✔️ | ✔️ | - |
| 403 | Sapotaceae | *Palaquium stenophyllum* H.J.Lam | LC | ✔️ | - | - | - | ✔️ | - | Rare-Sporadic | ✔️ | - | - | - |
| 404 | Sapotaceae | *Payena acuminata* Pierre | LC | - | ✔️ | ✔️ | - | ✔️ | - | Rare-Clustery | ✔️ | - | ✔️ | - |
| 405 | Sapotaceae | *Payena ferruginea* J.T.Pereira | LC | ✔️ | - | - | - | - | - | Rare-Clustery | ✔️ | - | - | - |
| 406 | Sapotaceae | *Payena lucida* A.DC. | NT | - | ✔️ | - | - | - | - | Rare-Clustery | ✔️ | - | ✔️ | - |
| 407 | Stemonuraceae | *Stemonurus malaccensis* (Mast.) Sleumer | - | - | - | - | - | - | - | Rare-Clustery | ✔️ | - | - | - |
| 408 | Styracaceae | *Bruinsmia styracoides* Boerl. & Koord.-Schum. | LC | - | - | - | - | - | - | Rare-Clustery | ✔️ | - | - | - |
| 409 | Symplocaceae | *Symplocos fasciculata* Zoll. | - | - | - | - | ✔️ | ✔️ | ✔️ | Rare-Clustery | ✔️ | ✔️ | ✔️ | - |
| 410 | Symplocaceae | *Symplocos paucinervia* Noot. | - | - | - | - | - | - | - | Rare-Clustery | ✔️ | - | - | - |
| 411 | Tetramelaceae | *Octomeles sumatrana* Miq. | LC | - | - | - | ✔️ | ✔️ | ✔️ | Rare-Sporadic | ✔️ | ✔️ | ✔️ | - |
| 412 | Theaceae | *Polyspora borneensis* (H.Keng) Orel, Peter G.Wilson, Curry & Luu | LC | ✔️ | - | - | - | - | - | Rare-Clustery | ✔️ | - | - | - |
| 413 | Theaceae | *Schima wallichii* (DC.) Korth. | LC | - | - | - | ✔️ | - | ✔️ | Rare-Sporadic | ✔️ | ✔️ | ✔️ | ✔️ |
| 414 | Theaceae | *Tetramerista glabra* Miq. | VU | - | - | - | - | ✔️ | ✔️ | Rare-Sporadic | ✔️ | ✔️ | - | - |
| 415 | Thymelaeaceae | *Aquilaria microcarpa* Baill. | VU | - | ✔️ | - | - | - | ✔️ | Rare-Sporadic | ✔️ | ✔️ | ✔️ | ✔️ |
| 416 | Thymelaeaceae | *Gonystylus affinis* Radlk. | Vu | - | - | - | - | - | - | Rare-Sporadic | ✔️ | - | - | - |
| 417 | Thymelaeaceae | *Gonystylus consanguineus* Airy Shaw | VU | ✔️ | - | - | - | - | - | Rare-Clustery | ✔️ | - | - | - |
| 418 | Thymelaeaceae | *Gonystylus macrophyllus* (Miq.) Airy Shaw | LC | - | ✔️ | - | - | - | - | Rare-Clustery | ✔️ | - | ✔️ | - |
| 419 | Urticaceae | *Dendrocnide elliptica* (Merr.) Chew | LC | - | - | - | - | - | ✔️ | Rare-Sporadic | ✔️ | ✔️ | - | - |
| 420 | Violaceae | *Rinorea lanceolata* (Roxb.) Kuntze | - | - | - | - | - | - | - | Rare-Clustery | ✔️ | - | - | - |
